# A Single *Streptomyces* Symbiont Makes Multiple Antifungals to Support the Fungus Farming Ant *Acromyrmex octospinosus*

**DOI:** 10.1371/journal.pone.0022028

**Published:** 2011-08-03

**Authors:** Ryan F. Seipke, Jörg Barke, Charles Brearley, Lionel Hill, Douglas W. Yu, Rebecca J. M. Goss, Matthew I. Hutchings

**Affiliations:** 1 School of Biological Sciences, University of East Anglia, Norwich Research Park, Norwich, United Kingdom; 2 Metabolic Biology, John Innes Centre, Norwich Research Park, Norwich, United Kingdom; 3 State Key Laboratory of Genetic Resources, and Evolution, Ecology, Conservation, and Environment Center, Kunming Institute of Zoology, Chinese Academy of Sciences, Kunming, China; 4 School of Chemistry, University of East Anglia, Norwich Research Park, Norwich, United Kingdom; University of Wisconsin – Madison, United States of America

## Abstract

Attine ants are dependent on a cultivated fungus for food and use antibiotics produced by symbiotic Actinobacteria as weedkillers in their fungus gardens. Actinobacterial species belonging to the genera *Pseudonocardia, Streptomyces* and *Amycolatopsis* have been isolated from attine ant nests and shown to confer protection against a range of microfungal weeds. In previous work on the higher attine *Acromyrmex octospinosus* we isolated a *Streptomyces* strain that produces candicidin, consistent with another report that attine ants use *Streptomyces*-produced candicidin in their fungiculture. Here we report the genome analysis of this *Streptomyces* strain and identify multiple antibiotic biosynthetic pathways. We demonstrate, using gene disruptions and mass spectrometry, that this single strain has the capacity to make candicidin and multiple antimycin compounds. Although antimycins have been known for >60 years we report the sequence of the biosynthetic gene cluster for the first time. Crucially, disrupting the candicidin and antimycin gene clusters in the same strain had no effect on bioactivity against a co-evolved nest pathogen called *Escovopsis* that has been identified in ∼30% of attine ant nests. Since the *Streptomyces* strain has strong bioactivity against *Escovopsis* we conclude that it must make additional antifungal(s) to inhibit *Escovopsis*. However, candicidin and antimycins likely offer protection against other microfungal weeds that infect the attine fungal gardens. Thus, we propose that the selection of this biosynthetically prolific strain from the natural environment provides *A. octospinosus* with broad spectrum activity against *Escovopsis* and other microfungal weeds.

## Introduction

Insect fungiculture has been best studied in the attine ants (subfamily Myrmicinae, tribe Attini) whose common ancestor is estimated to have evolved agriculture around 50 million years ago [1]. Attine ants are so dependent on the cultivation of fungus that when a daughter queen leaves to found a new nest she carries a piece of the cultivar fungus in her mouth in order to establish a culture of that fungus in her new nest [1]. Intriguingly, attine ants have also evolved a mutualism with Actinobacteria that produce antibiotics that the ants use as weedkillers to keep their fungal gardens free of other microbes [2], [3], [4]. The relationship between Actinobacteria, fungal cultivar and attine ant has been intensely studied in the branch of higher attines known as the leaf-cutting ants (genera *Atta* and *Acromyrmex*) which harvest fresh vegetation to feed to their highly specialised fungal cultivar, *Leucoagaricus gongylophorus* [1]. The fungus has evolved lipid and carbohydrate rich hyphae known as gongylidia which the ants harvest and use as food [5]. Pathogens of the fungal garden, most notably fungi of the genus *Escovopsis*, if left unchecked, can destroy a fungal garden and lead to the collapse of the colony within weeks [6], [7].

Two overlapping but conflicting theories have been put forward to explain the evolution of mutualism between attine ants and Actinobacteria. The first suggests co-evolution of attine ants and Actinobacteria belonging to the genus *Pseudonocardia*. This theory suggests that the fungus garden pathogen *Escovopsis* has also co-evolved and that *Pseudonocardia* and *Escovopsis* are engaged in an evolutionary arms race in which the bacteria evolve compounds that specifically target *Escovopsis* but do not inhibit the growth of the fungal cultivar [5]. The second model suggests that attine ants select antifungal-producing Actinobacteria from the environment and is consistent with the identification of additional Actinobacterial genera on leaf-cutting ants, including *Streptomyces* and *Amycolatopsis* species [3], [4], [8], [9]. However, these theories are not mutually exclusive and evidence suggests attine ants co-evolve with *Pseudonocardia* bacteria and still select other antifungal producing bacteria from the soil, perhaps to prevent evolution of resistance in the fungal pathogens [9], [10]. Indeed, there is good evidence, both direct and indirect, that leaf-cutting ants use multiple antifungals produced by multiple Actinobacteria during the cultivation of their fungal gardens suggesting that both models do apply, at least in the higher attine genus *Acromyrmex* [3], [11]. Recent work demonstrated that *Acromyrmex octospinosus* are associated with a *Pseudonocardia* strain that may have co-evolved and a *Streptomyces* strain that was most likely acquired from the environment relatively recently [9].

One intriguing question that still needs to be addressed in the environmental recruitment model concerns how the ants select beneficial bacteria. Actinobacteria are well known producers of useful secondary metabolites, including around 60% of all known antibiotics [12] and it seems likely that this production capability is key to their success as mutualists. Clearly the production of antifungals makes them useful to the ants and we hypothesise that production of multiple antifungals with different targets by single Actinobacterial species would make them more attractive to the ants as mutualists. In this work we carry out a more in depth analysis of the antifungals made by one of the strains associated with the leaf-cutting ant *A. octospinosus*, a species of *Streptomyces* which has been proposed to support fungus growing ants through production of the polyene antifungal candicidin [4], [9]. We report the genome sequence and analysis of this strain indicating its capacity to make numerous antibiotics, including at least three antifungal compounds. Curiously, this strain makes both of the antifungals that have been reported in the *Streptomyces* attine ant mutualists but neither the candicidin or antimycins are obligatory for the inhibition of the co-evolved nest pathogen *E. weberi*. We propose that additional and as yet unknown antifungal(s) made by this strain specifically target *Escovopsis* and that candicidin and antimycins offer protection against other microfungal weeds. We propose that the ability of this single species to make multiple antifungal compounds makes it an attractive acquisition for the ants and their fungiculture.

## Results

### Genome sequencing and analysis

To determine the antibiotic biosynthetic capability of *Streptomyces* S4 a combination of shotgun, 3 kbp and 8 kbp paired end libraries were constructed and 454 pyrosequenced to generate >335 Mbp of sequence that was assembled into 12 scaffolds containing 211 large contigs. The genome consists of one ∼7.5 Mbp linear chromosome, which is within the size range reported for genomes of other sequenced streptomycetes, as well as one linear plasmid (∼180 kbp) and one circular plasmid (∼2 kbp) [13]. The *Streptomyces* S4 genome was annotated by a combination of manual and automated methods and multiple biosynthetic gene clusters predicted to produce secondary metabolites were identified. Table 1 summarizes characteristics of predicted natural product biosynthetic gene clusters in *Streptomyces* S4. As expected based on our previous work, the *Streptomyces* S4 genome contained a candicidin biosynthetic gene cluster that shares 98% nucleotide sequence identity with the candicidin biosynthetic gene cluster from *Streptomyces* sp. FR-008 [14]. Other biosynthetic gene clusters of note are a non-ribosomal peptide synthetase (NRPS) biosynthetic gene cluster that is predicted to direct the biosynthesis of an antibacterial similar to mannopeptimycin, as well as a NRPS biosynthetic gene cluster whose predicted product is a gramicidin-like antibacterial. We note these antibacterials could be useful in eliminating competition during colonisation of the ant cuticle. *Streptomyces* S4 also contains a type II polyketide synthase (PKS) biosynthetic gene cluster that shares 100% nucleotide identity to that of the fredericamycin biosynthetic gene cluster characterized in *S. griseus* [15]. Fredericamycin is mostly known for its antitumor properties [16]. Additionally, there are six functionally unassigned biosynthetic gene clusters (three NRPS and three hybrid NRPS/PKS) that are not similar to gene clusters with known products. It is likely that at least some these clusters encode secondary metabolites with antibacterial or antifungal activity.

**Table 1:** Putative secondary metabolites encoded by *Streptomyces* S4.

Predicted biosynthetic system	Genome coordinates	Predicted metabolite or close relative	Biological properties
Hopene / squalene synthase	scaffold08: 588141–598581	Hopanoids	Membrane stabilizers
NRPS-independent siderophore synthetase	scaffold05: 959198–972403	Desferrioxamine	Siderophore
NRPS-independent siderophore synthetase	scaffold08: 1448607–1457963	Unknown	Unknown
Ectoine synthase	scaffold05: 68880–72152	Ectoine	Osmolyte
Phytoene / polyprenyl synthase	scaffold06: 410147–419826	Carotenoids	Pigment
Terpene synthase	scaffold08: 1719586–1721871	Geosmin	Unknown
Type III PKS	scaffold06: 295706–300701	1,3,6,8-tetrahydroxynaphthalene	Pigment
Type I PKS	scaffold06: 115150–253654	Candicidin	Antifungal
Type I PKS / Type III PKS	scaffold05:1001127–1064995	Kendomycin	Anticancer
Type II PKS	scaffold08: 3878554–3911349	Fredericamycin	Anticancer
Hybrid NRPS / PKS	scaffold06: 81953–106578	Unknown	Unknown
Hybrid NRPS / PKS	scaffold06: 7264–45109	Unknown	Unknown
Hybrid NRPS / PKS	scaffold08: 503983–520001	Unknown	Unknown
NRPS	scaffold08: 4240081–4309220	Gramicidin	Antibacterial
NRPS	scaffold08: 3002155–3042863	Mannopeptimycin	Antibacterial
NRPS	scaffold06: 65083–81878	Unknown	Unknown
NRPS	scaffold08: 276268–301035	Unknown	Unknown
NRPS	scaffold08: 3930113–3950474	Unknown	Unknown

NRPS, non-ribosomal peptide synthetase, PKS, polyketide synthase.

### Mutagenesis of the candicidin biosynthetic gene cluster does not abolish antifungal activity

**Figure 1:**
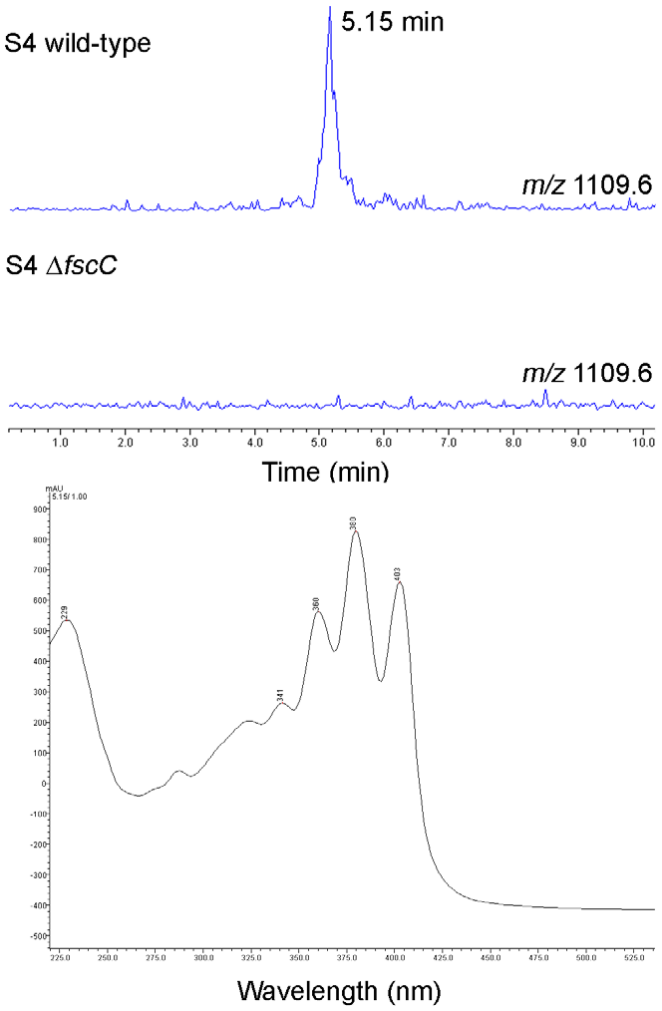
Deletion of the candicidin biosynthetic gene, *fscC* abolishes production of candicidin. LC-MS was used to analyze supernatant from *Streptomyces* S4 wild-type and S4 Δ*fscC*. The extracted ion chromatogram for candicidin (*m/z* 1109.6) is shown and confirmed that only S4 wild-type and not the Δ*fscC* mutant produced candicidin. The UV visible spectra for the peak at RT 5.15 min displays absorption characteristics consistent with polyene compounds is also shown (bottom).

**Figure 2:**
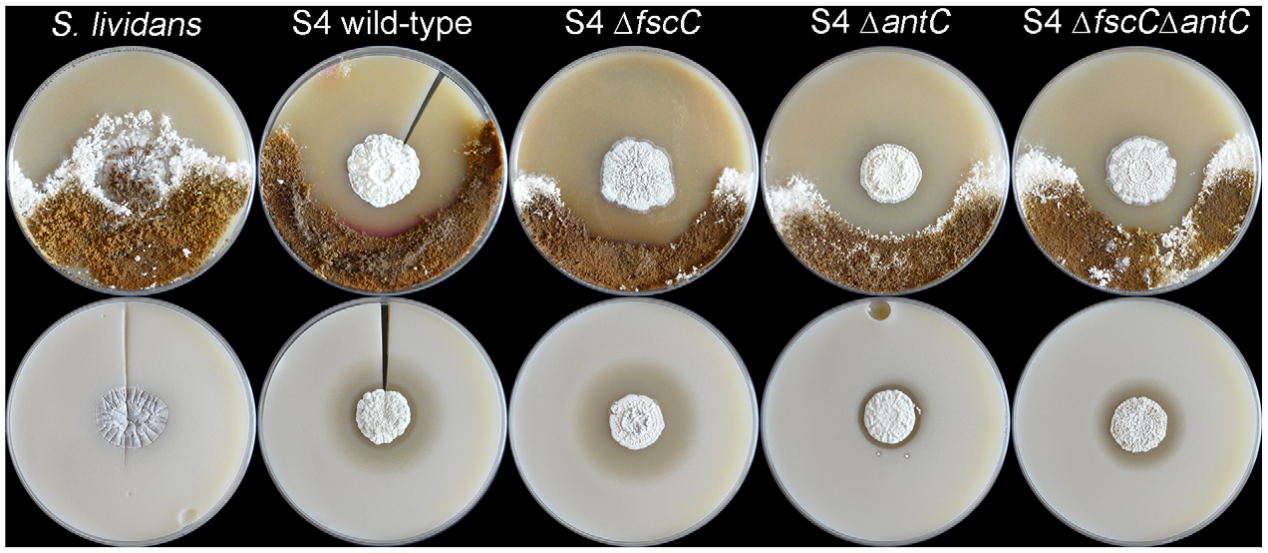
Antifungal bioactivity of the non-antifungal-producing strain *Streptomyces lividans*, *Streptomyces* S4 wild-type and mutant strains. Bioassays with *Streptomyces lividans*, S4 wild-type, S4 Δ*fscC*, S4 Δ*antC* and S4 Δ*fscC* Δ*antC* against *Escovopsis weberi* (top panel) and *C. albicans* (bottom panel) demonstrate that deletion of *fscC* does not abolish antifungal activity and that deletion of *antC* only reduces antifungal activity against *C. albicans* and not *E. weberi*. The S4 Δ*fscC* Δ*antC* double mutant does not display reduced antifungal activity against *E. weberi* suggesting the presence of an additional antifungal compound that is responsible for the phenotype observed during in vitro bioassays.

Evidence that candicidin is not the sole antifungal generated by *Streptomyces* S4 is demonstrated by the retained antifungal activity of a mutant strain deficient in the biosynthesis of this compound. The candicidin biosynthetic gene cluster was disrupted by deletion of the polyketide synthase gene, *fscC*, which encodes the candicidin biosynthetic modules 6–10 [14]. LC-MS analysis of butanol-extracted culture supernatants from the wild-type strain revealed a molecular ion (*m/z* 1109.6) consistent with that of candicidin D and showed characteristic polyene absorption bands in its UV spectrum, with absorbance maxima at 360, 380, 403 nm (Fig. 1). As predicted, the molecular ion *m/z* 1109.6 was not detected in the Δ*fscC* mutant, indicating that candicidin production is abolished in this strain (Fig. 1). Bioassays of the isogenic wild-type and Δ*fscC* strains against *C. albicans* and the nest pathogen *Escovopsis weberi* demonstrated that loss of candicidin has no effect on the antifungal bioactivity of *Streptomyces* S4 (Fig. 2). This result suggests that *Streptomyces* S4 makes at least one additional antifungal compound that has not been identified previously, most likely encoded by one of the other biosynthetic gene clusters identified in this work.

### Identification of the antimycin biosynthetic gene cluster

**Figure 3:**
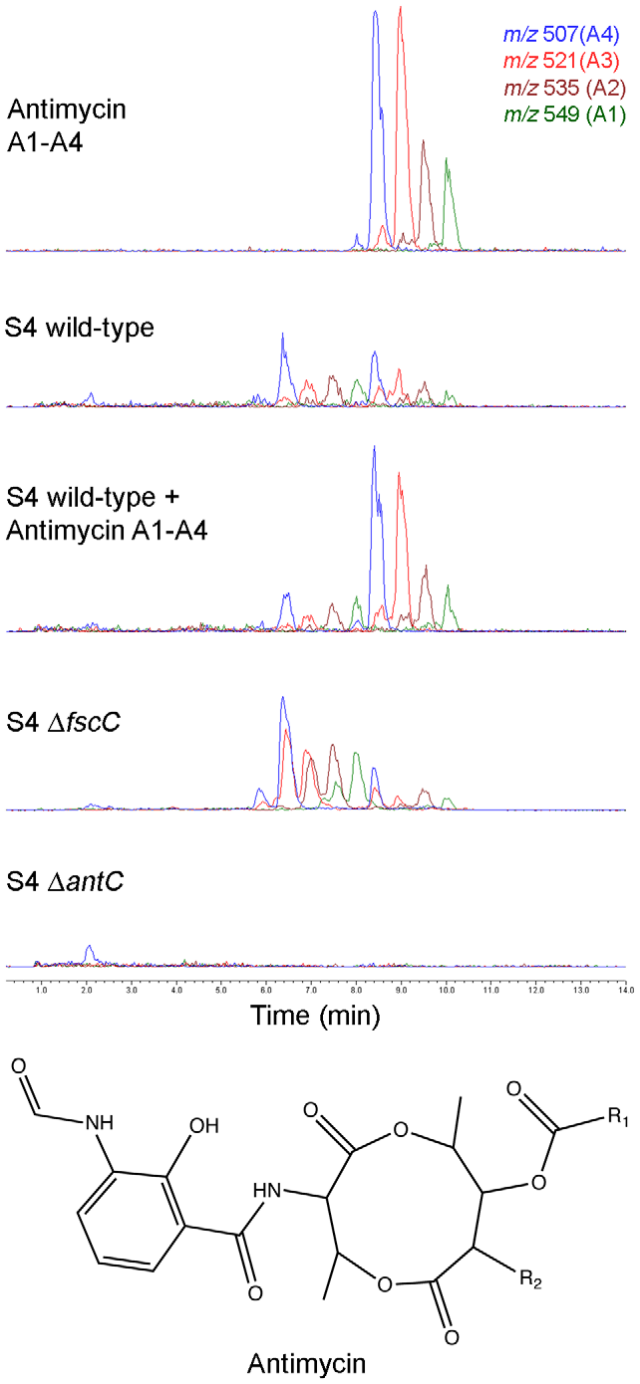
LC/MS analysis of *Streptomyces* S4 wild-type and mutant strains compared to antimycin standards. The extracted ion chromatograms for antimcyins A1–A4 are shown. Eight compounds consistent with the mass of antimycin A1–A4 were produced by S4 wild-type and S4 Δ*fscC*, but were not produced by the Δ*antC* mutant. Co-injection of antimycin A1–A4 with the S4 wild-type extract demonstrated that antimycin A1–A4 have the same retention time as four of the eight compounds produced by S4 wild-type. The UV visible spectra and ESI positive mode mass spectra for antimycin A1–A4 and the eight antimycin compounds produced by S4 wild-type are shown in Fig. S1 and Fig. S2, respectively. Antimycin A1: R_1_ = CH(CH_3_)CH_2_CH_3_, R_2_ = (CH_2_)_5_CH_3_. Antimycin A2: R_1_ = CH(CH_3_)_2_, R_2_ = (CH_2_)_5_CH_3_. Antimycin A3: R_1_ = CH(CH_3_)CH_2_CH_3_, R_2_ = (CH_2_)_3_CH_3_. Antimycin A4: R1 = CH(CH_3_)_2_ R_2_ = (CH_2_)_3_CH_3_.

While this work was in progress another group reported that antimycins are produced by a number of the other *Streptomyces* strains associated with attine ant nests [17]. Antimycins inhibit the respiratory chain and are known to have antifungal activity. We investigated whether *Streptomyces* S4 is making antimycin compounds in addition to candicidin and hypothesised that antimycins could potentially account for the retained bioactivity against *Escovopsis* observed for the *Streptomyces* S4 *fscC* mutant. LC-MS analysis of culture supernatants of the wild-type strain identified eight compounds with *m/z* that match those reported for antimycins A1–A4 (Fig. 3). To determine if any of the eight compounds could be antimycins, we co-injected commercially available antimycin standards A1–A4 with our wild-type extract, which revealed that four of the eight compounds possess the same retention time as the antimycin A1–A4 standards (Fig. 3). Four of the eight S4 compounds were identical to the commercially available antimycin standards A1–A4 both in terms of UV absorbance profile and LC retention time. Whilst the remaining four S4 compounds possess the same UV absorbance characteristics as the antimycin standards (Fig. S1), and the same *m/z* parent ions as those of the standards, they exhibit different retention times (Fig. 3); further experiments are being carried out to identify and characterize these four previously unreported compounds.

**Figure 4:**
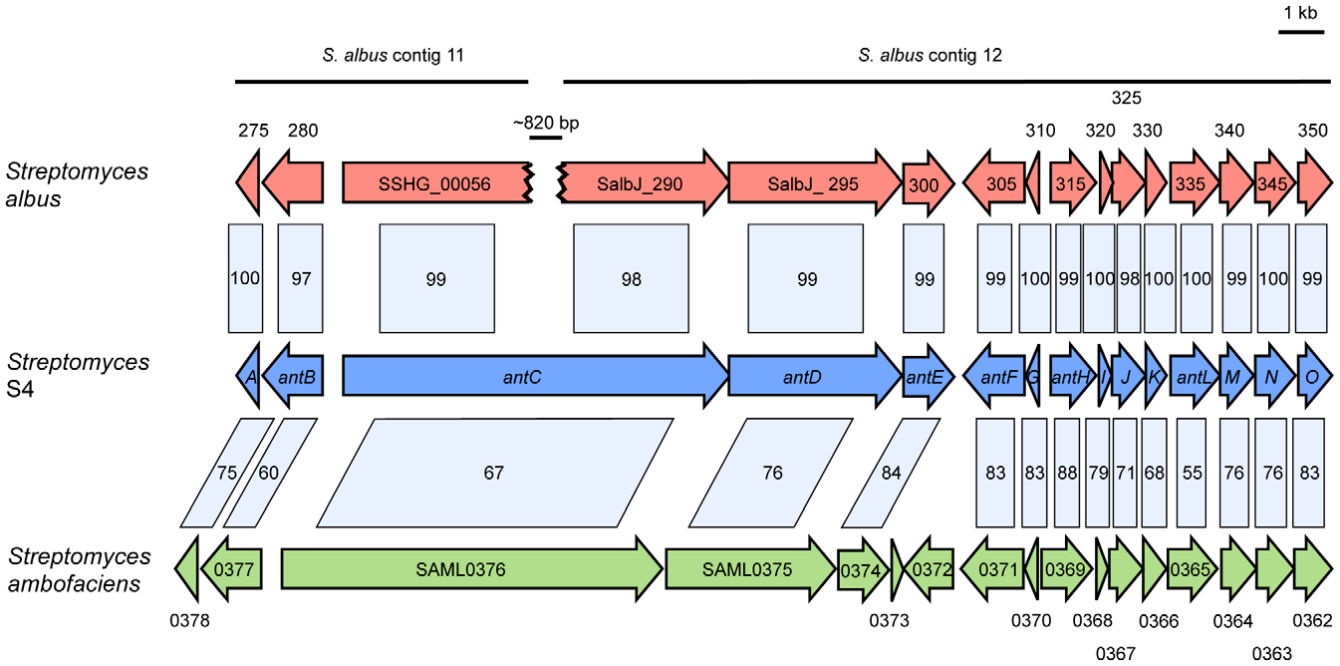
Gene schematic of the *Streptomyces* S4 antimycin biosynthetic gene cluster and comparison to putative antimycin clusters in *Streptomyces albus* and *Streptomyces ambofaciens*. The percent amino acid homology shared between S4 proteins and proteins in *S. albus* and *S. ambofaciens* is indicated in the shaded boxes. The draft genomic sequence of *S. albus* is incomplete and the sequence for the putative antimycin biosynthetic gene cluster is split over contig 11 and contig 12 with an estimated gap of ∼820 bp in the *antC* gene. The *Streptomyces* S4 antimycin biosynthetic gene cluster is located on scaffold06 at coordinates 81953–106578. The partial genome sequences of *Streptomyces* S4, *S. albus* J1074, and *S. ambofaciens* ATCC 23877 are available under accession numbers CADY00000000, ABYC00000000 and AM238663, respectively. The gene names for *S. albus* have been shortened to eliminate the first nine numbers of the gene name (e.g. SalbJ_290 = SalbJ_010100000290).

To our knowledge the gene cluster that encodes the antimycin biosynthetic pathway has not been identified, despite these compounds first being isolated over 60 years ago [18]. The structure of antimycin suggests that it may be synthesized, at least in part, by an NRPS, and that threonine may be utilized as a substrate (Fig. 3). The Basic Local Alignment Search Tool [19] revealed a region of the *Streptomyces* S4 genome with 57% amino acid sequence identity to the threonine adenylation domain from the daptomycin biosynthetic protein, DptA [20]. This region of homology enabled us to identify a hybrid NRPS/PKS biosynthetic gene cluster that displays significant amino acid identity to a hybrid NRPS/PKS biosynthetic gene cluster present in both *S. albus* and *S. ambofaciens* and potentially encodes for the biosynthesis of antimycins (Fig. 4). Table 2 displays the proposed functions of proteins present in the hybrid NRPS/PKS cluster. In order to determine if this biosynthetic gene cluster can direct the production of antimycin, we disrupted the hybrid NRPS/PKS gene, *antC* and assessed antifungal activity against *C. albicans* in a plate bioassay. The Δ*antC* mutant displayed dramatically reduced antifungal activity against *C. albicans* compared to that of the wild-type strain (Fig. 2). This strongly suggested that the product of this cluster was an antifungal compound and is consistent with the hypothesis that this cluster could potentially mediate the biosynthesis of antimycins, compounds known to possess strong antifungal activity against *C. albicans* (Fig. 2). Confirmation that the hybrid NRPS/PKS encoded by *Streptomyces* S4 directs the biosynthesis of antimycins was obtained by comparing the LC-MS profiles of the wild-type, Δ*fscC* and Δ*antC* mutant strains. Extracted ion chromatograms revealed that the Δ*antC* mutant does not produce the eight antimycins and that the Δ*fscC* mutant retained the ability to produce these antimycin compounds (Fig. 3).

**Table 2:** Proposed functions of proteins encoded by the antimycin biosynthetic gene cluster.

*Streptomyces* S4 protein	Proposed function
AntA	Sigma factor
AntB	Condensation domain
AntC	Non-ribosomal peptide synthetase
AntD	Polyketide synthase
AntE	Dehydrogenase
AntF	Acyl-CoA ligase
AntG	Thiolation domain
AntH	Phenylacetate dioxygenase
AntI	Phenylacetate dioxygenase
AntJ	Phenylacetate dioxygenase
AntK	Phenylacetate dioxygenase
AntL	Oxidoreductase
AntM	Dehydrogenase
AntN	Tryptophan 2,3-dioxygenase
AntO	Lipase

### A *Streptomyces* S4 mutant which cannot make candicidin or antimycins still has antifungal activity against *E. weberi*

Our bioassays against *E. weberi* with the Δ*fscC* and Δ*antC* mutant strains of *Streptomyces* S4 did not show a reduction in bioactivity against this nest parasite indicating that key antifungal compound(s) affording protection against the natural fungal pathogen still remain to be identified (Fig. 2). To determine whether additional antifungals are made by *Streptomyces* S4 we generated a new mutant in which the *fscC* and *antC* genes were disrupted in the same strain and assessed the bioactivity of this strain against *C. albicans* and *E. weberi*. As we predicted, the antifungal activity of the Δ*fscC* Δ*antC* double mutant against *E. weberi* was comparable to that of the wild-type strain, confirming that additional antifungal compound(s) made by *Streptomyces* S4 account for the majority of the antifungal activity observed against *E. weberi* in vitro. The additional antifungal(s) may be encoded by one of the five functionally unassigned biosynthetic gene clusters identified in our genome analysis (Table 1) or by a gene cluster not identified in our analysis.

## Discussion

Although the attine ant-fungal mutualism has been studied for more than a century, the antibiotic-producing Actinobacterial mutualists were only discovered ∼15 years ago and it is only very recently that scientists have started to address the nature of the antibiotics being produced by these bacteria [2], [4], [9], [17]. It has been hypothesised, although not proven, that these antibiotics are used by the ants to kill off contaminated parts of the garden and / or to suppress the growth of fungal pathogens including co-evolved pathogens in the genus *Escovopsis* and many other microfungal weeds [6], [7]. Recent studies have shown that strains belonging to two key genera are typically associated with attine ants, species of *Pseudonocardia*, which have been suggested to have co-evolved with the ants and to be transmitted vertically by the queens, and species of *Streptomyces* which have been suggested to be more recently acquired from the environment [3], [4], [8], [9]. Two antifungals have been identified from proposed mutualist species in each genus and both inhibit the nest pathogen *Escovopsis* in vitro. *Pseudonocardia* associated with the lower attine *A. dentigerum* makes dentigerumycin and *Pseudonocardia* associated with the higher attine *A. octospinosus* makes nystatin P1 [2], [9]. *Streptomyces* mutualists associated with higher attines of the genus *Acromyrmex* are known to produce the well-known antifungals candicidin and antimycins and it has been suggested that these compounds account for the bioactivity of this *Streptomyces* strain against *Escovopsis* [4], [9], [17]. To date almost all of this work has been carried out through isolation and mass spectrometry analysis of the antifungal compounds, although we used genome scanning of a *Pseudonocardia* mutualist to identify a nystatin-like biosynthetic gene cluster and its product which we named nystatin P1 [9].

In this work we have undertaken the first in-depth genome sequence analysis of a proposed attine ant mutualist, in this case a candicidin-producing *Streptomyces* strain isolated from *A. octospinosus* garden worker ants collected in Trinidad [9]. Genome sequencing and analysis identified 17 gene clusters that are predicted to encode for known or unknown secondary metabolites, including the known gene cluster for candicidin biosynthesis. Following the discovery of antimycin production by *Streptomyces* strains isolated from attine ants in a separate study [17] we identified a gene cluster encoding a pathway that is consistent with antimycin biosynthesis. Surprisingly, despite antimycins first being isolated and characterised >60 years ago the antimycin biosynthetic pathway was not known. We identified eight compounds which we assigned as antimycins and then identified and disrupted the hybrid NRPS/PKS gene cluster which we predicted to encode antimycin biosynthesis. The production of the eight antimycin compounds was abolished in the mutant strain providing strong evidence that this gene cluster does indeed encode the antimycin biosynthetic pathway.

In bioassays of the wild-type *Streptomyces* S4 strain alongside strains which cannot make candicidin, antimycins or either of these antifungal compounds we found that whilst antimycin- and candicidin-deficient strains had reduced activity against the human pathogen *C. albicans* their activity against the nest pathogen *E. weberi* was unaltered. This is curious as the *Streptomyces* S4 strain and *E. weberi* are thought to have co-evolved in this mutualism [21]. This suggests that despite previous research demonstrating that purified candicidin and antimycin preparations inhibit the growth of *Escovopsis* in vitro neither compound is responsible for the activity observed in bioassays where *Streptomyces* S4 is challenged with *E. weberi*. We conclude that these compounds potentially inhibit the growth of other microfungal weeds found in the ant-fungus gardens while additional and currently unknown antifungal(s) produced by *Streptomyces* S4 have stronger activity against *Escovopsis*. We also propose that the combination of antifungals produced by this single *Streptomyces* strain coupled with the antifungal(s) produced by a *Pseudonocardia* strain isolated from the same nest provides a broad spectrum of antifungal activity that is used by the ants to farm their fungus. Furthermore, the antibacterials made by *Streptomyces* S4 potentially help it to outcompete other bacteria for the ant host.

Our data suggest that the *Streptomyces* strains isolated by other researchers from attine ants are likely to make additional antifungals since they appear to be closely related to *Streptomyces* S4. It will be important to re-examine the biosynthetic capability of these strains in order to fully understand the chemical basis of their interactions with attine ants and their fungal cultivar. This reflects a common problem in the field of natural product antibiotic discovery, in which the reisolation of known compounds hampers the discovery of new antibiotics. The approach we have outlined here is time consuming and technically challenging, but it is perhaps the only way to determine the entire biosynthetic capability of an antibiotic-producing strain particularly if some of the antibiotics being made, and their biosynthetic gene clusters, are new to science. Future work will be aimed at determining the products of the five unassigned biosynthetic gene clusters in *Streptomyces* S4 and identifying the additional antifungal compound(s) made by this strain. This is likely to involve significant challenges if, as we predict, these are novel secondary metabolites.

In conclusion, although good progress has been made recently we are still a long way from understanding the chemical basis of the symbioses between antibiotic-producing Actinobacteria and their attine ant hosts. We hope that our study will stimulate further research in this area and the identification of additional antifungal and antibacterial compounds in this system.

## Materials and Methods

### Growth media and strains

*Streptomyces* strains were routinely grown on soya flour mannitol (SFM) agar plates or in liquid TSB/YEME while *E. coli* strains were grown on Lysogeny both- Lennox (LB) [22]. Media was supplemented with antibiotics as required at the following concentrations: carbenicillin (100 µg/ml), hygromycin B (50 µg/ml), nalidixic acid (25 µg/ml), apramycin (50 µg/ml). S4 was isolated and identified by 16S rDNA sequencing in a previous study (GenBank accession HM179229). Antifungal bioassays with *C. albicans* and *E. weberi* were carried out as described previously [9]. Strains and plasmids are described in Table 3.

**Table 3:** Strains and plasmids used in this study.

Strain or plasmid	Genotype or comments	Source or reference
*Streptomyces* S4	Wild type	[9]
*Streptomyces* S4 Δ*fscC*	S4 *fscC* null mutant	This study
*Streptomyces* S4 Δ*antC*	S4 *antC* disruption mutant	This study
*Streptomyces* S4 Δ*fscC ΔantC*	S4 *fscC* and *antC* double knockout strain	This study
*Candida albicans*	*Candidia albicans* CA-6	[26]
*Escovopsis weberi*	*Escovopsis weberi* (CBS 11060)	[9]
*E. coli* ET12567	Non-methylating host for transfer of DNA into *Streptomyces* spp. (*dam*, *dcm*, *hsdM*)	[25]
*E. coli* TOP10	Host for routine cloning procedures	Invitrogen
Plasmids		
pGEMT-EZ	Cloning vector for PCR products; Amp^R^	Promega
pIJ773	Source of the *aac(3)*IV+oriT apramycin resistance resistance marker	[27]
pIJ10700	PCR template for *hygR* cassette	[24]
pUZ8002	Encodes conjugation machinery for mobilization of plasmids from *E. coli* to *Streptomyces*; Kan^R^	[25]
pKC1132	Suicide vector used for constructing gene deletions in *Streptomyces* spp. Apr^R^ and contains conjugal origin of transfer	[23]
pKC1132-Up	Derivative of pKC1132 containing the *fscC* upstream knockout arm cloned into the HindIII and BamHI restriction sites	This study
pKC1132-UpDn	Derivative of pKC1132-Up containing the *fscC* downstream knockout arm cloned into the BamHI and EcoRI restriction sites	This study
pKC1132-UpHygDn	Derivative of pKC1132-UpDn containing the hygromycin resistance cassette from pIJ10700 cloned into the BamHI site	This study
pGEMT-Ant	Derivative of pGEMT-EZ containing the a 1.5 kb fragment of the *antC* gene, Amp^R^	This study
pGEMT-AntApr	Derivative of pGEMT-Ant containing the *aac(3)*IV+oriT apramycin resistance gene from pIJ773 cloned into the BamHI site provided by RFS121	This study

Amp^R^, ampicillin resistance, Apr^R^, apramycin resistance, Hyg^R^, hygromycin resistance, Kan^R^, kanamycin resistance, *oriT*, origin of transfer.

### Construction of *Streptomyces* S4 mutant strains

In order to create the Δ*fscC* mutant, two 3 kb knockout arms were PCR amplified using GoTaq Polymersae (Promega) with oligonucleotide primers RFS78 and RFS79 (upstream arm) and RFS80 and RFS81 (downstream arm), respectively. Oligonucleotide primers (Integrated DNA Technologies) were engineered at their 5′ end to contain restriction sites for cloning (Table S1). The resulting PCR products were cloned into pGEMT-EZ (Promega) and sequenced to verify their identity. The upstream arm was released from pGEMT-EZ with HindIII and BamHI and cloned into pKC1132 (which contained the RK2 conjugal origin of transfer and as well as an apramycin resistance gene [22], [23] cut with the same enzymes to result in pKC1132-Up. Next, the downstream arm was released from pGEMT-EZ with BamHI and EcoRI and cloned into pKC1132-Up cut with the same enzymes to result in pKC1132-UpDn. Finally, a hygromycin B resistance cassette was PCR-amplified from pIJ10700 [24] using oligonucleotides RFS94 and RFS95 engineered to contain BamHI sites at their 5′ end. The hygromycin resistance cassette was cloned into pGEMT-EZ and subsequently released by BamHI digestion and cloned into pKC1132-UpDn cut with the same enzyme to result in pKC1132-UpHygDn.

The plasmid pKC1132-UpHygDn was electroporated into *E. coli* strain ET12567/pUZ8002 [25] and transferred to *Streptomyces* S4 by cross-genera conjugation as previously described [22]. Transconjugants were selected for apramycin resistance. An apramycin-resistant transconjugant was obtained and subsequently replica plated to obtain hygromycin-resistant and apramycin-sensitive colonies, a phenotype indicating that the *fscC* gene had been entirely replaced by the hygromycin resistance cassette and that the plasmid backbone was no longer present. Loss of the pKC1132-UpHygDn plasmid backbone and mutagenesis of the *fscC* gene in the Δ*fscC* strain was confirmed by PCR.

In order to disrupt the *antC* gene a ∼1.5 kb internal fragment of the *antC* gene was PCR amplified using oligonucleotide primers RFS121 and RFS122 which were engineered to contain BamHI and EcoRI restriction sites at their 5′ end, respectfully (Table S1). The resulting PCR product was sequenced to verify its identity and cloned into pGEMT-EZ to result in pGEMT-Ant. The apramycin resistance cassette containing a conjugal origin of transfer (*aac(3)*IV+oriT) was isolated from pIJ773 as a BamHI fragment and cloned into the BamHI site (provided by RFS121) in pGEMT-Ant to result in pGEMT-AntApr. The pGEMT-AntApr plasmid was electroporated into ET12567/pUZ8002 and mobilized to S4 wild-type and S4 Δ*fscC* by conjugation. Transconjugants were selected for apramycin resistance, a phenotype indicating that the disruption plasmid has crossed into the chromosome. Disruption of the *antC* gene was confirmed by PCR amplification using oligonucleotide primers (RFS147 and M13F, and RFS148 and M13R) targeting the DNA sequence upstream and downstream of the expected site of integration.

### LC-MS analysis

*Streptomyces* S4 wild-type and mutant strains were cultivated in mannitol-soya flour liquid medium in a 250 ml flask shaking at 270 rpm. For analysis of candidicin production, cultures were harvested after 10 days of growth, bacterial cells were removed by centrifugation and the supernatant of three biological replicates was combined. Fifty milliliters of the combined supernatant was extracted three times with an equal volume of butanol. Butanol extracts were combined and evaporated to dryness under vacuum and the residue was resuspended in 0.5 ml of 50% aqueous methanol. For analysis of antimycin production, cultures were harvested after 4 days of growth, bacterial cells were removed by centrifugation and the supernatant of two biological replicates was combined and extracted with XAD16 resin (Sigma). Following extraction, the resin was washed twice with ten milliliters of deionized water and *Streptomyces* S4 metabolites were eluted from the resin with one milliliter of 100% methanol. Prior to LC-MS analysis the methanol elution was diluted with water to a final methanol content of 50%. Antimycin A1–A4 standards were purchased from Sigma Aldrich. Immediately prior to LC-MS analysis samples were spun in a microcentrifuge at maximum speed for 5 minutes to remove insoluble material. Only the supernatant (10 µl) was used for injection into a Shimadzu single quadrupole LC-MS-2010A mass spectrometer equipped with Prominence HPLC system as described previously [9]. For co-injection of antimycin A1-A4 with S4 wild-type extract 5 µl of standard and 5 µl of wild-type extract were mixed immediately prior to injection into the LC-MS.

## Supporting Information

Figure S1 **UV absorbance spectra for antimycin A1–A4 and eight antimycin compounds produced by** ***Streptomyces*** **S4.** The UV absorbance spectra is shown for A) antimycin A4 (RT = 8.40), B) antimycin A3 (RT = 8.97), C) antimycin A2 (RT = 9.50), D) antimycin A1 (RT = 10.00), E) S4 metabolite 1 (RT = 6.38), F) S4 metabolite 2 (RT = 6.87), G) S4 metabolite 3 (RT = 7.43), H) S4 metabolite 4 (RT = 8.00), I) S4 antimycin A4 (RT = 8.40), J) S4 antimycin A3 (RT = 8.97), K) S4 antimycin A2 (RT = 9.50), L) S4 antimycin A1 (RT = 10.00).
(PDF)

Figure S2 **Mass spectra for antimycin A1–A4 and eight antimycin compounds produced by** ***Streptomyces*** **S4.** The ESI positive mode detection mass spectra is shown for A) antimycin A4 (RT = 8.40), B) antimycin A3 (RT = 8.97), C) antimycin A2 (RT = 9.50), D) antimycin A1 (RT = 10.00), E) S4 metabolite 1 (RT = 6.38), F) S4 metabolite 2 (RT = 6.87), G) S4 metabolite 3 (RT = 7.43), H) S4 metabolite 4 (RT = 8.00), I) S4 antimycin A4 (RT = 8.40), J) S4 antimycin A3 (RT = 8.97), K) S4 antimycin A2 (RT = 9.50), L) S4 antimycin A1 (RT = 10.00).
(PDF)

Table S1 Oligonucleotide primers used in this study.
(DOC)

## References

[1] Schultz TR, Brady SG (2008). Major evolutionary transitions in ant agriculture.. Proc Natl Acad U S A.

[2] Oh DC, Poulsen M, Currie CR, Clardy J (2009). Dentigerumycin: a bacterial mediator of an ant-fungus symbiosis.. Nat Chem Biol.

[3] Sen R, Ishak HD, Estrada E, Dowd SE, Hong E (2009). Generalized antifungal activity and 454-screening of *Pseudonocardia* and *Amycolatopsis* bacteria in nests of fungus-growing ants.. Proc Natl Acad U S A.

[4] Haeder S, Wirth R, Herz H, Spiteller D (2009). Candicidin-producing *Streptomyces* support leaf-cutting ants to protect their fungus garden against the pathogenic fungus *Escovopsis*.. Proc Natl Acad U S A.

[5] Currie CR (2001). A community of ants, fungi, and bacteria: a multilateral approach to studying symbiosis.. Annu Rev Microbiol.

[6] Reynolds HT, Currie CR (2004). Pathogenicity of *Escovopsis weberi*: the parasite of the attine ant-microbe symbiosis direclty consumes the ant-cultivated fungus.. Mycologia.

[7] Rodrigues A, Bacci M, Mueller UG, Ortiz A, Pagnocca FC (2008). Microfungal ‘weeds’ in the leafcutter ant symbiosis.. Microb Ecol.

[8] Kost C, Lakatos T, Bottcher I, Arendholz W-R, Redenbach M (2007). Non-specific association between filamentous bacteria and fungus-growing ants.. Naturwissenschaften.

[9] Barke J, Seipke RF, Grüschow S, Heavens D, Drou N (2010). A mixed community of actinomycetes produce multiple antibiotics for the fungus farming ant *Acromyrmex octospinosus*.. BMC Biol.

[10] Barke J, Seipke RF, Yu DW, Hutchings MI (2011). A mutualistic microbiome: how do fungus-growing ants select their antibiotic-producing bacteria.. Commun Integr Biol.

[11] Mueller UG, Dash D, Rabeling C, Rodrigues A (2008). Coevolution between attine ants and actinomycete bacteria: a reevaluation.. Evolution.

[12] Challis GL, Hopwood DA (2003). Synergy and contingency as driving forces for the evolution of multiple secondary metabolite production by *Streptomyces* species.. Proc Natl Acad U S A.

[13] Seipke RF, Crossman L, Drou D, Heavens D, Bibb MJ (2011). Draft genome sequence of *Streptomyces* S4, a symbiont of the leafcutter ant *Acromyrmex octospinosus*.. J Bacteriol.

[14] Chen S, Huang X, Zhou X, Bai L, He J (2003). Organizational and mutational analysis of a complete FR-008/candicidin gene cluster encoding a structurally related polyene complex.. Chem Biol.

[15] Wendt-Pienkowski E, Huang Y, Zhang J, Li B, Jiang H (2005). Cloning, sequencing, analysis, and heterologous expression of the fredericamycin biosynthetic gene cluster from *Streptomyces griseus*.. J Am Chem Soc.

[16] Pandey RC, Toussaint MW, Stroshane RM, Kalita CC, Aszalos AA (1981). Fredericamycin A, a new antitumor antibiotic I. production, isolation and physicochemical properties.. J Antibiot.

[17] Schoenian I, Spiteller M, Ghaste M, Wirth R, Herz H (2011). Chemical basis of the synergism and antagonism in microbial communities in the nests of leaf-cutting ants.. Proc Natl Acad U S A.

[18] Dunshee BR, Leben C, Keitt GW, Strong FM (1949). The isolation and properties of antimycin A.. J Am Chem Soc.

[19] Altschul SF, Gish W, Miller W, Myers EW, Lipman DJ (1990). Basic local alignment search tool.. J Mol Biol.

[20] Miao V, Coeffet-LeGal M-F, Brian P, Brost R, Penn J (2005). Daptomycin biosynthesis in *Streptomyces roseosporus*: cloning and analysis of the gene cluster and revision of peptide stereochemistry.. Microbiol.

[21] Currie CR, Wong B, Stuart AE, Schultz TR, Rehner A (2003). Ancient tripartite coevolution in the attine ant-microbe symbiosis.. Science.

[22] Kieser T, Bibb MJ, Buttner MJ, Chater KF, Hopwood DA (2000). Practical *Streptomyces* Genetics.

[23] Bierman M, Logan R, O'Brien K, Seno ET, Rao RN (1992). Plasmid cloning vectors for the conjugal transfer of DNA from *Escherichia coli* to *Streptomyces* spp.. Gene.

[24] Gust B, Chandra G, Jakimowicz D, Yuqing T, Bruton CJ (2004). Lambda Red-mediated genetic manipulation of antibiotic-producing *Streptomyces*.. Adv Appl Microbiol.

[25] MacNeil DJ, Gewain KM, Ruby CL, Dezeny G, Gibbons PH (1992). Analysis of *Streptomyces avermitilis* genes required for avermectin biosynthesis utilizing a novel integrative vector.. Gene.

[26] Maconi P, Bistoni F, Boncio A, Boncio L, Bersiani A (1976). Utilizzazione di una soluzione salina ipertonica di cloruro di potassio (3M KCl) per l'estrazione di antigeni solubili da Candida albicalns.. Ann Sclavo.

[27] Gust B, Challis GL, Fowler K, Kieser T, Chater KF (2003). PCR-targeted *Streptomyces* gene replacement identifies a protein domain needed for biosynthesis of the sesquiterpene soil odor geosmin.. Proc Natl Acad U S A.

