# Supplementary figures and images for: A Single Streptomyces Symbiont Makes Multiple Antifungals to Support the Fungus Farming Ant Acromyrmex octospinosus
Source: PLoS One. 2011 Aug 3;6(8):e22028. doi: 10.1371/journal.pone.0022028 (PMC3153929; doi:10.1371/journal.pone.0022028)

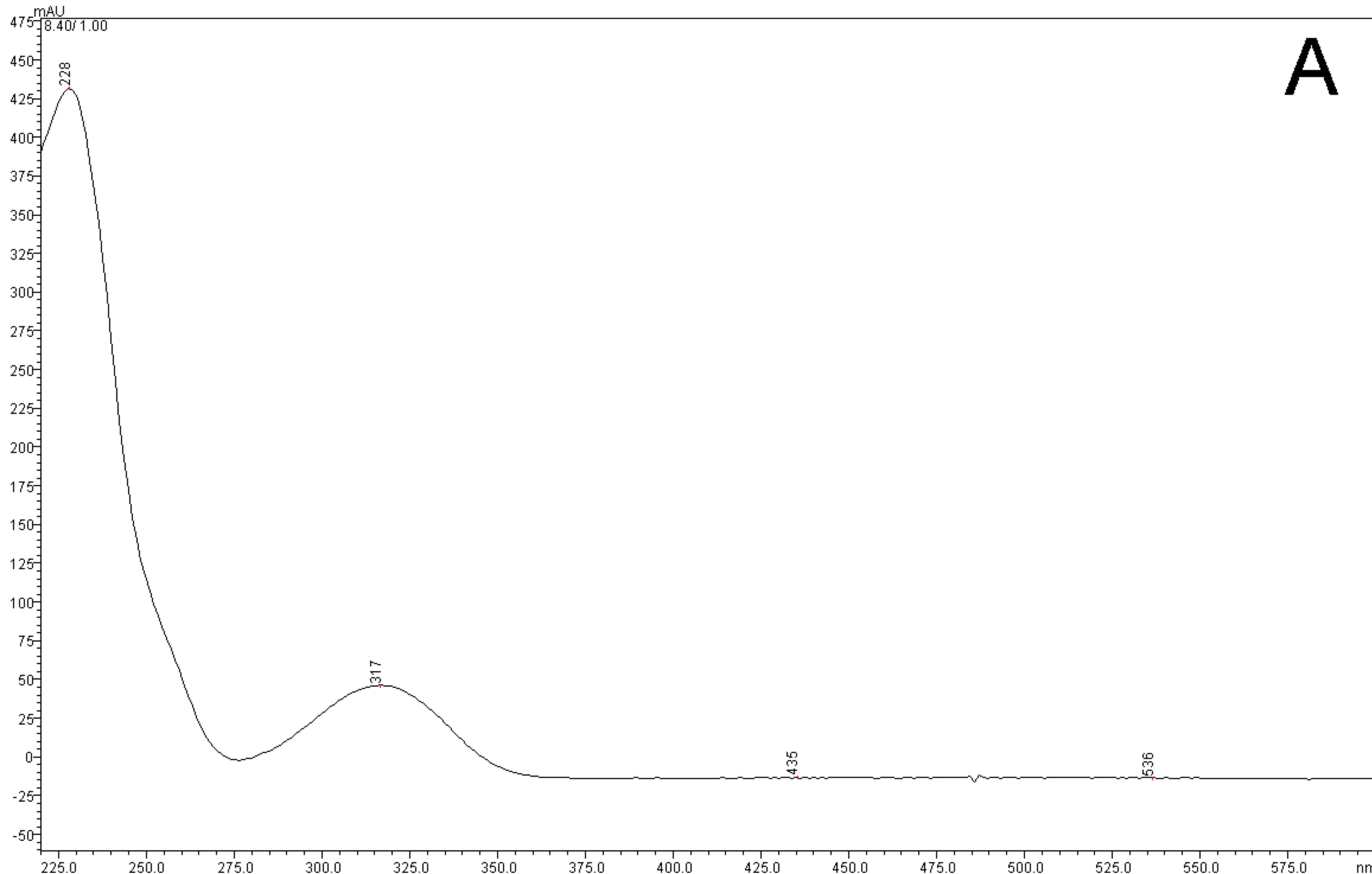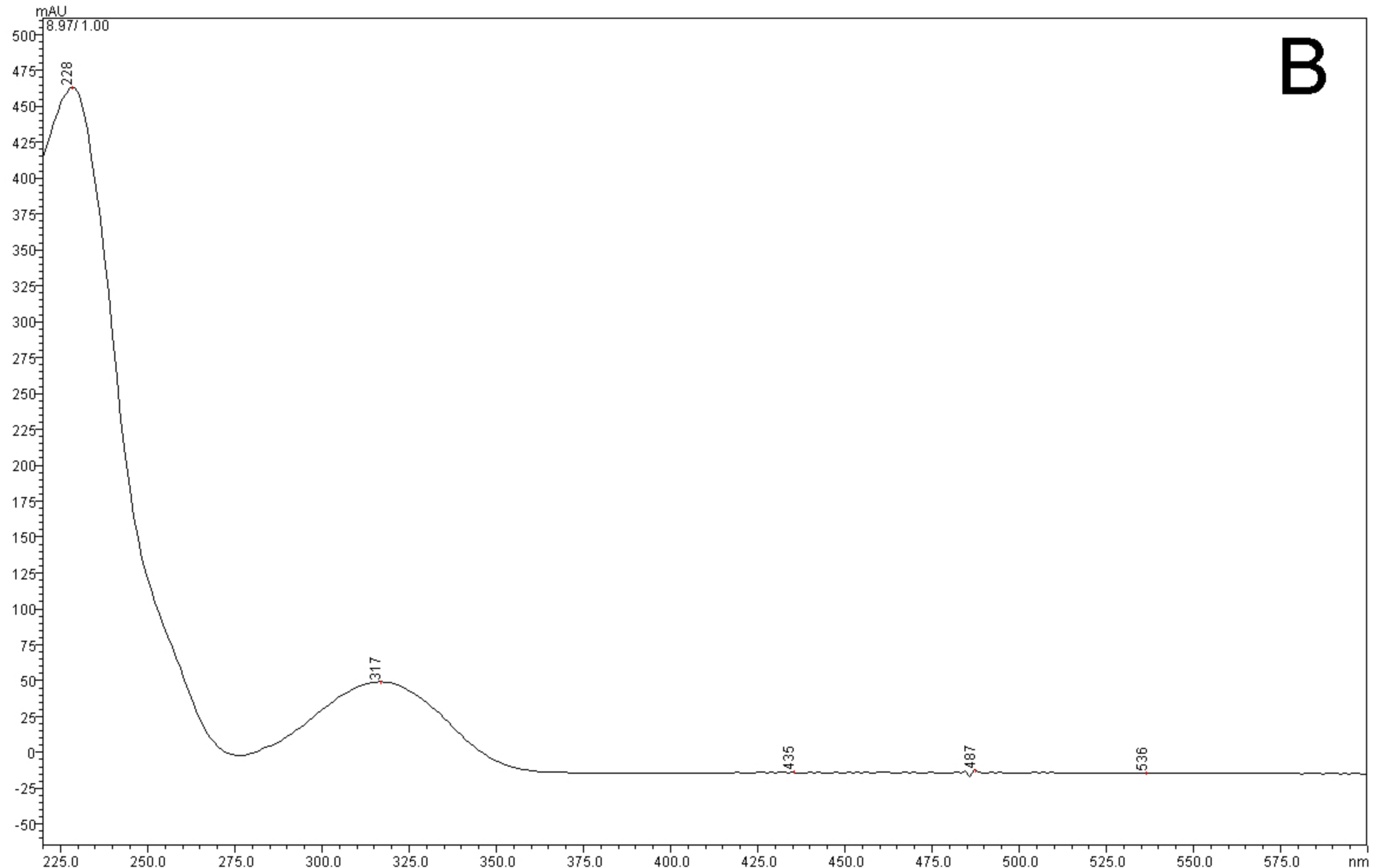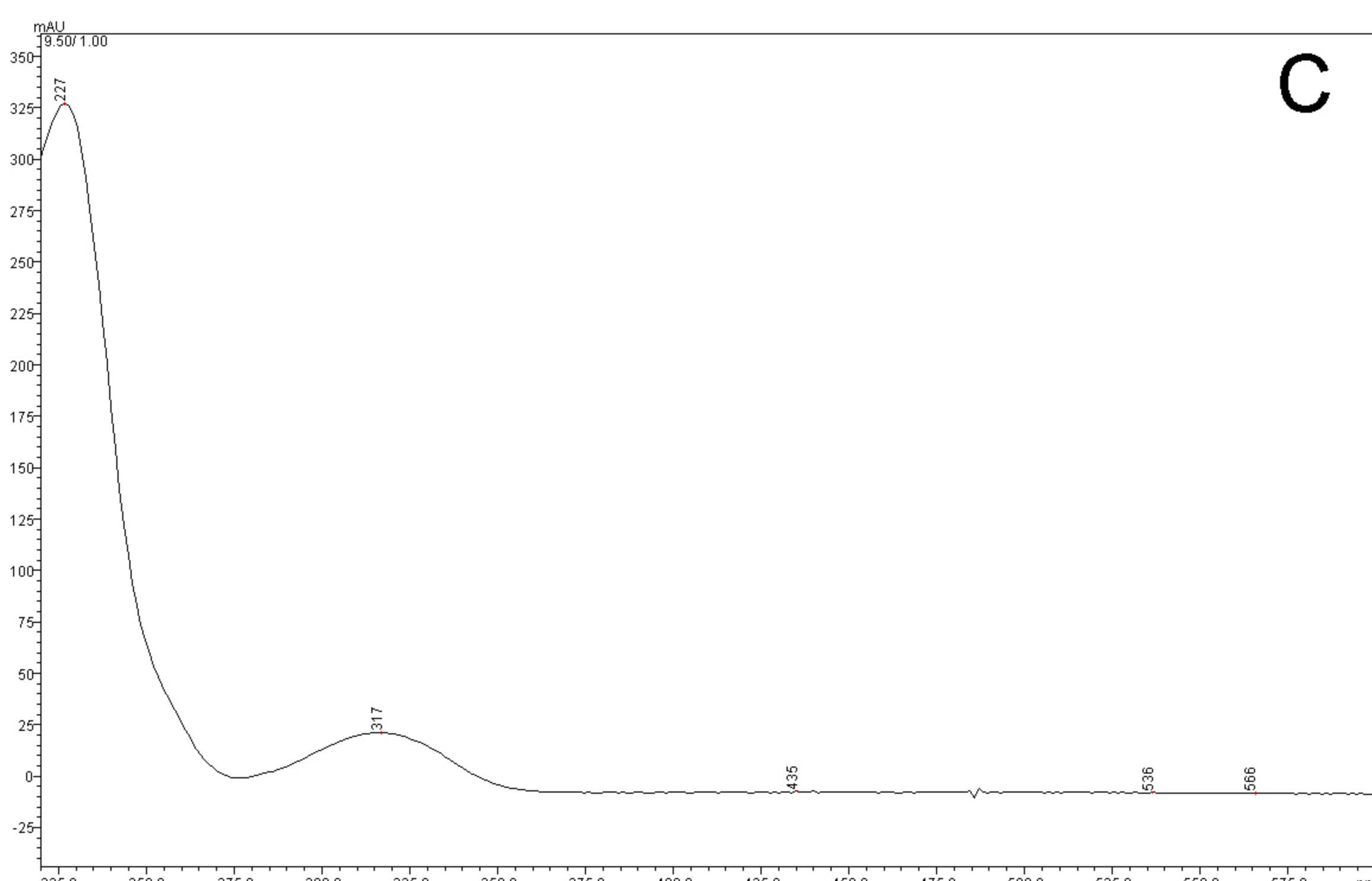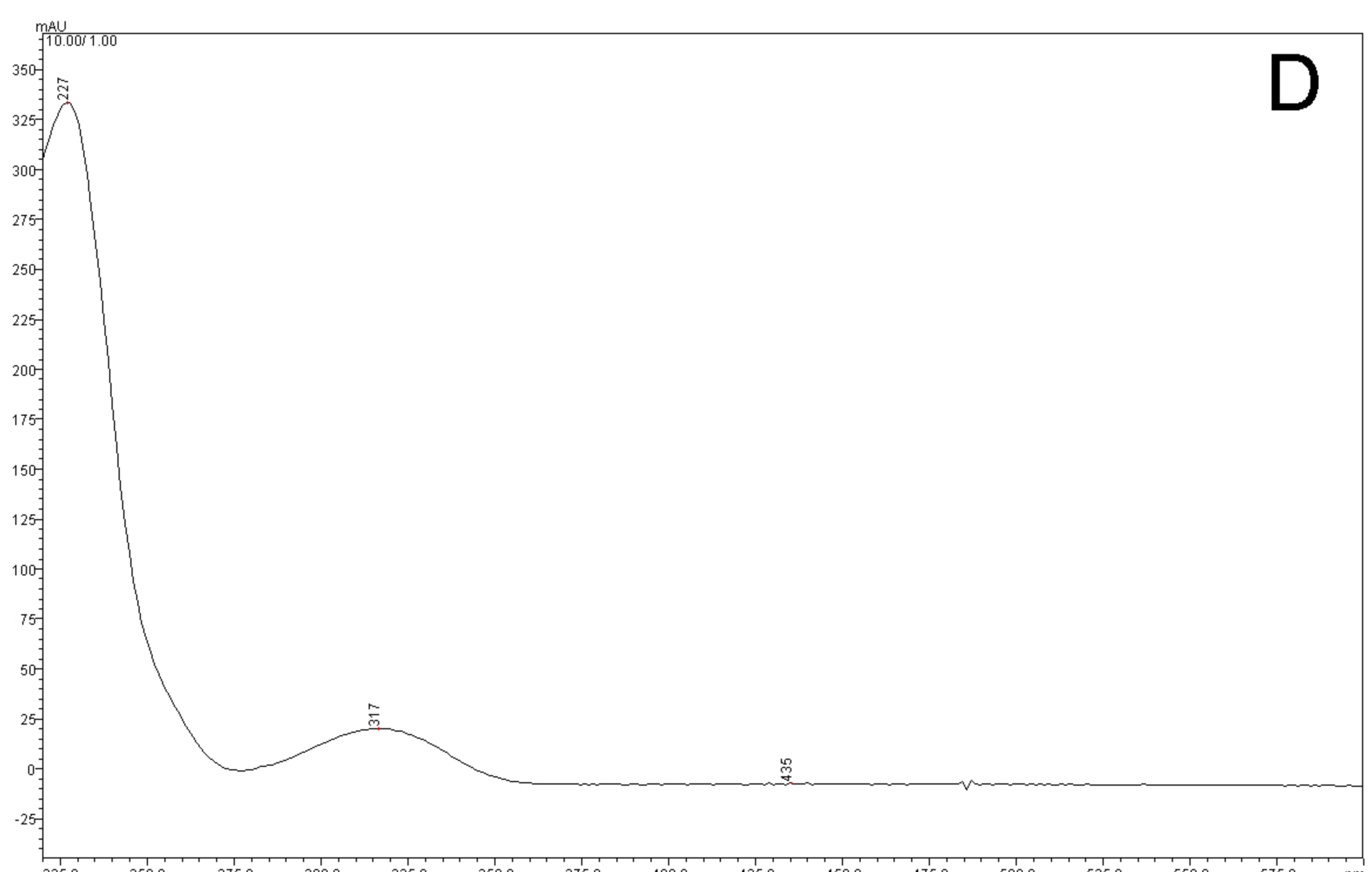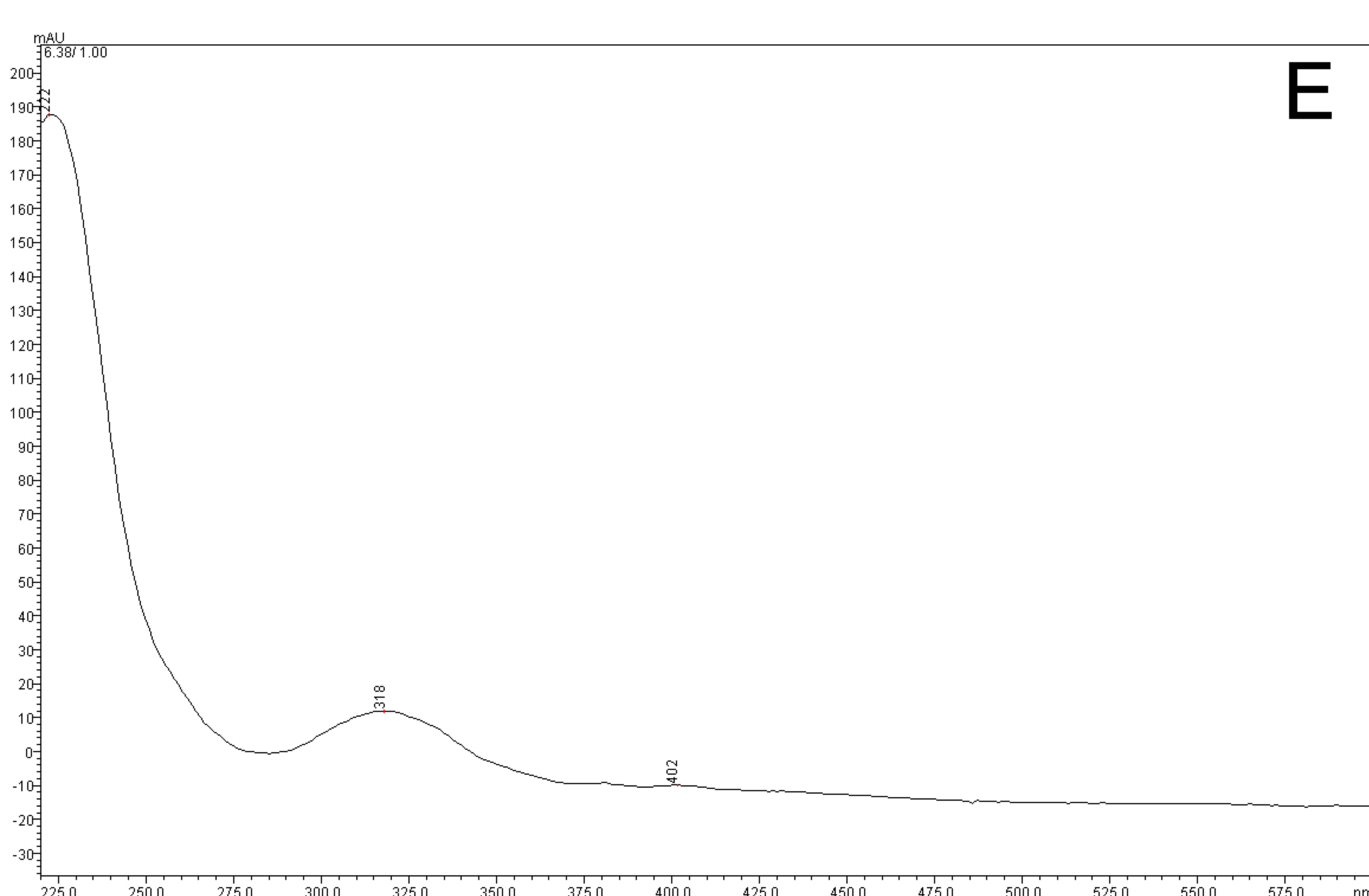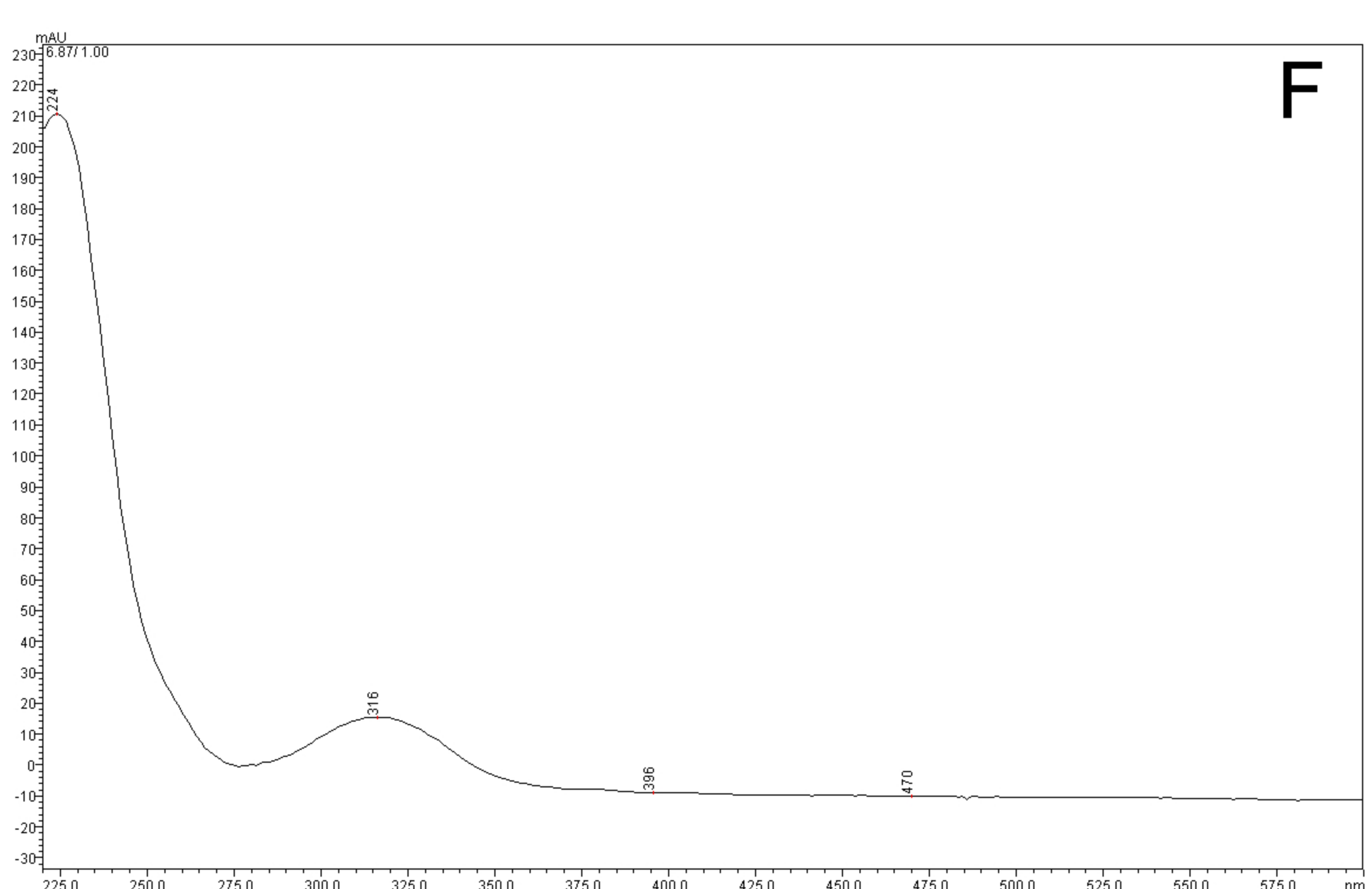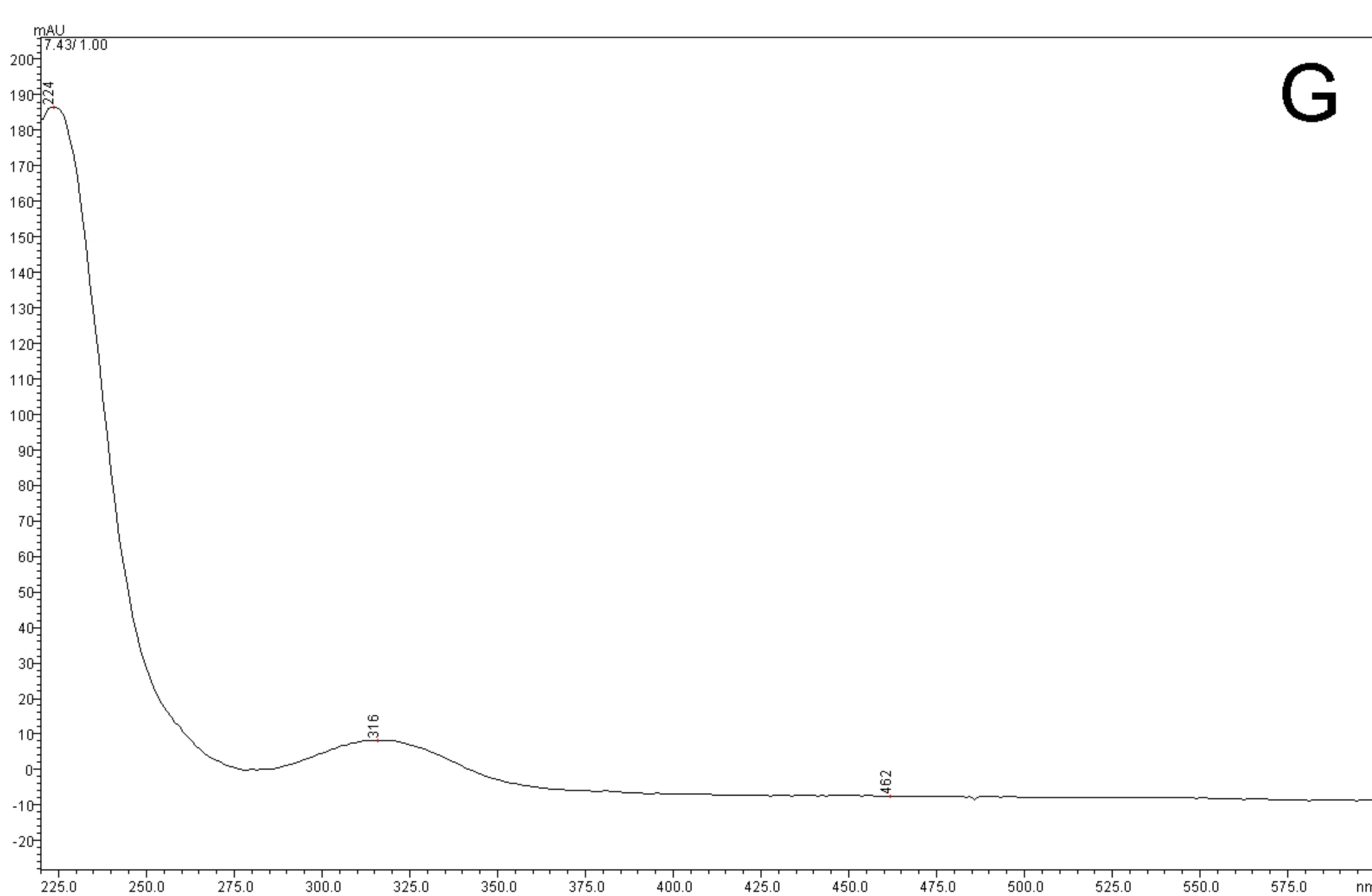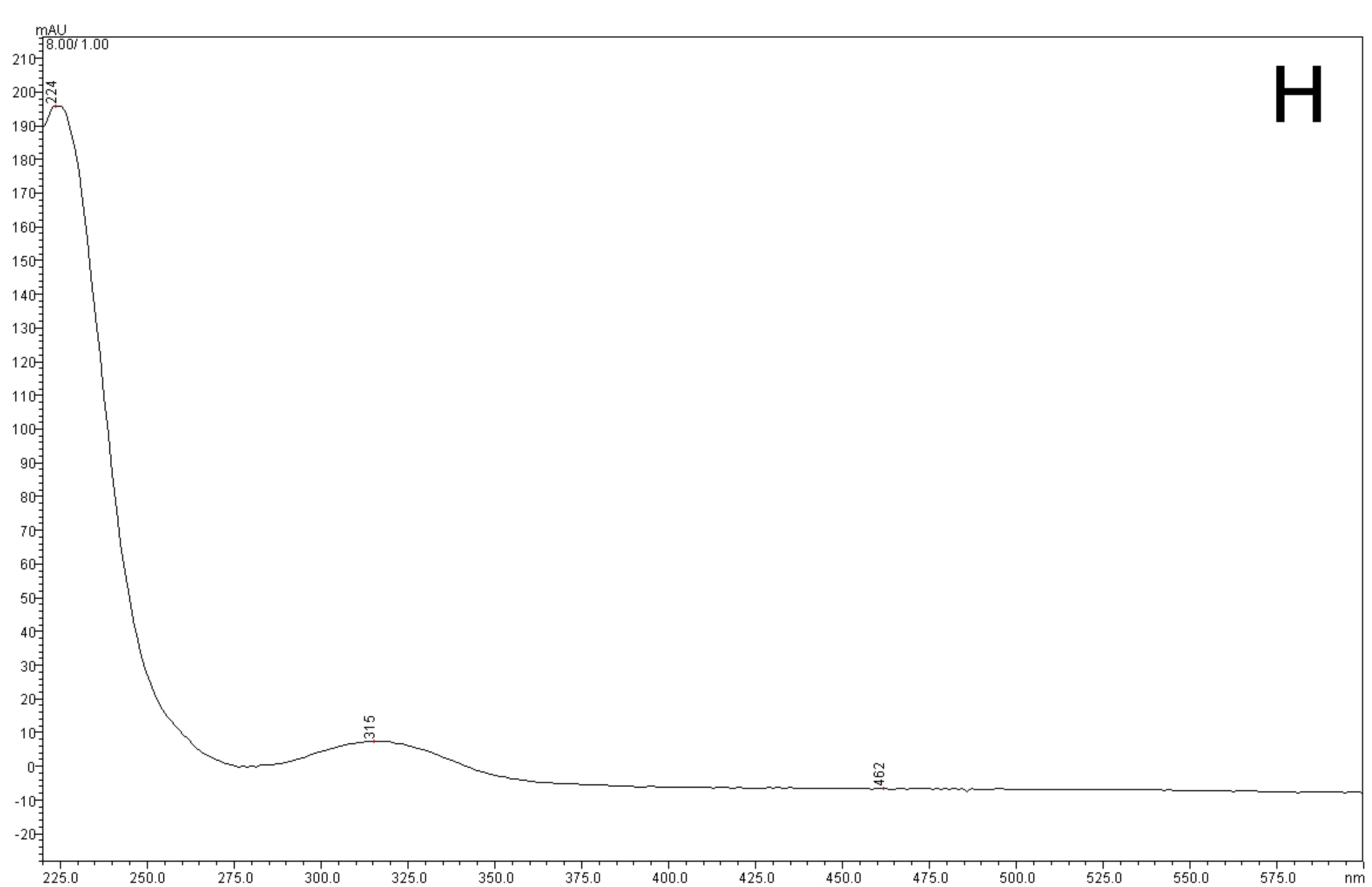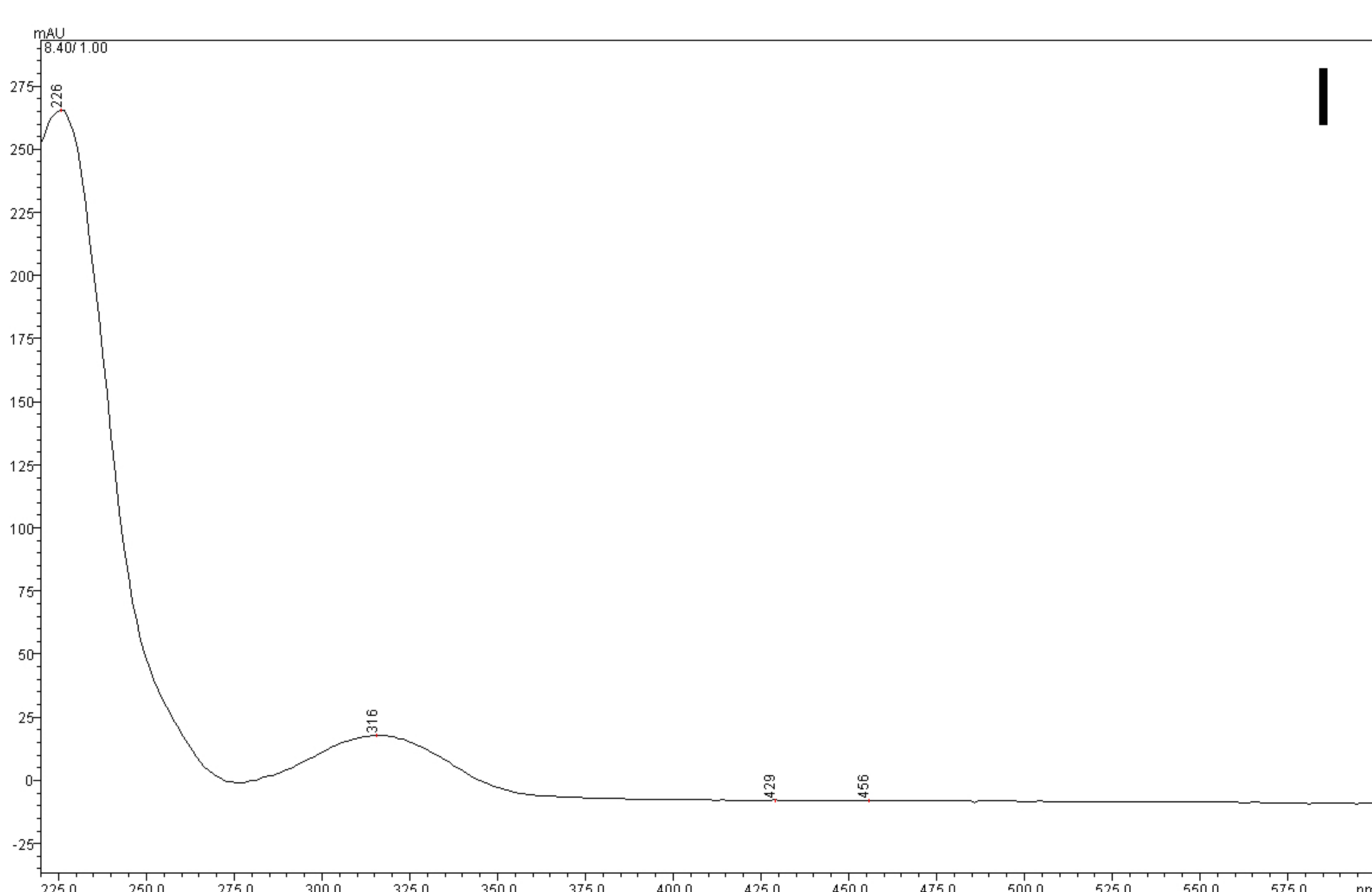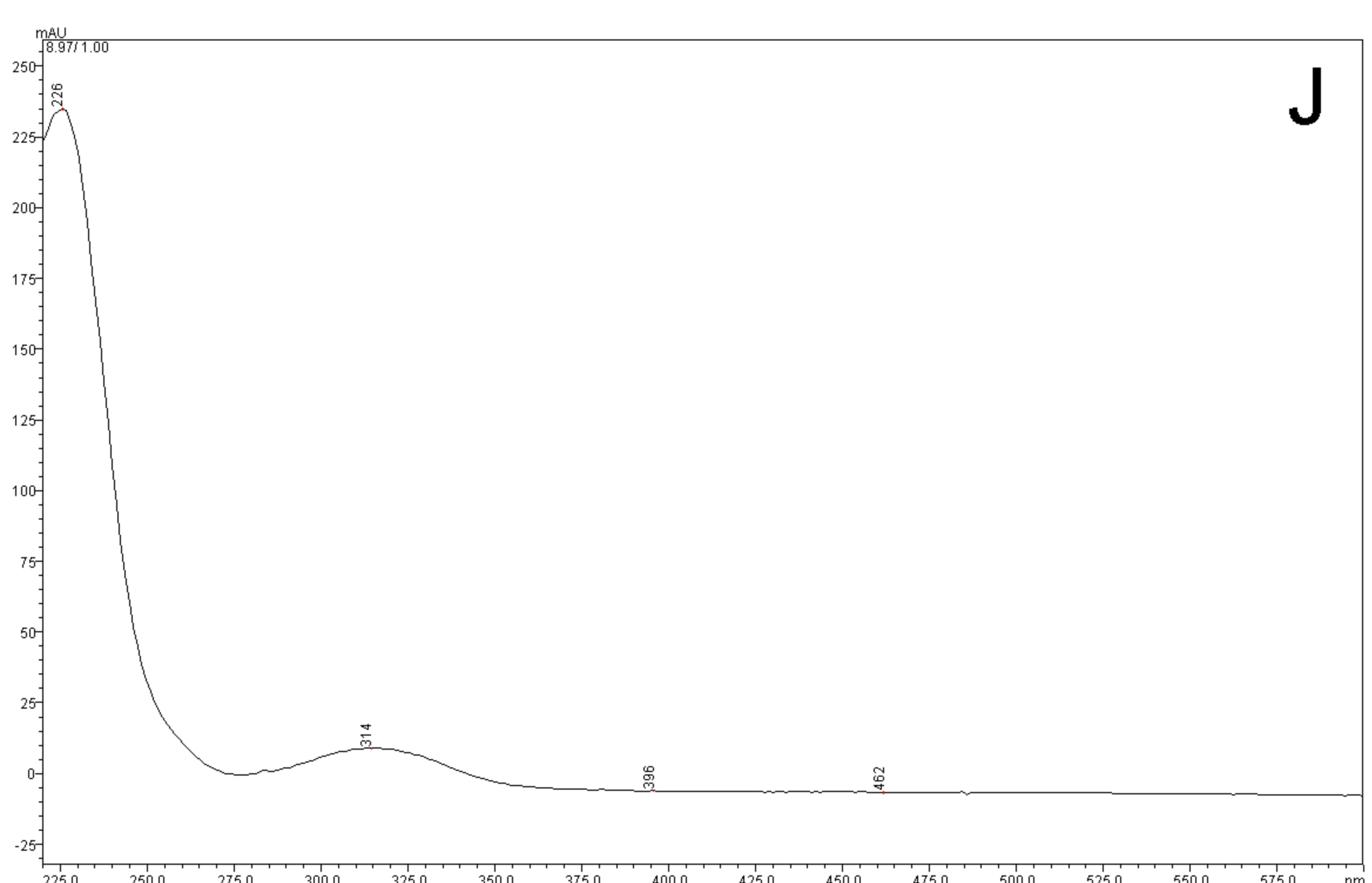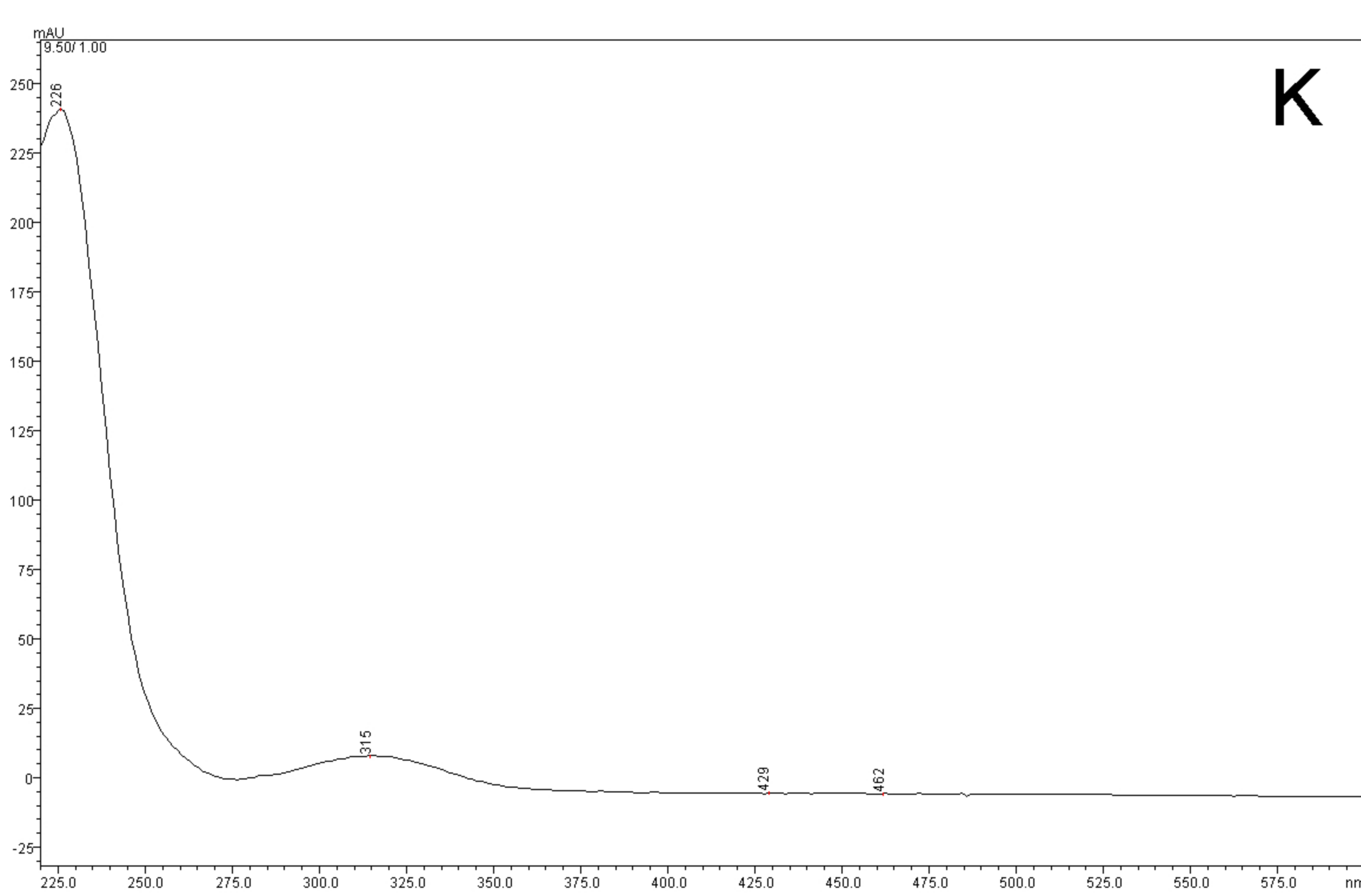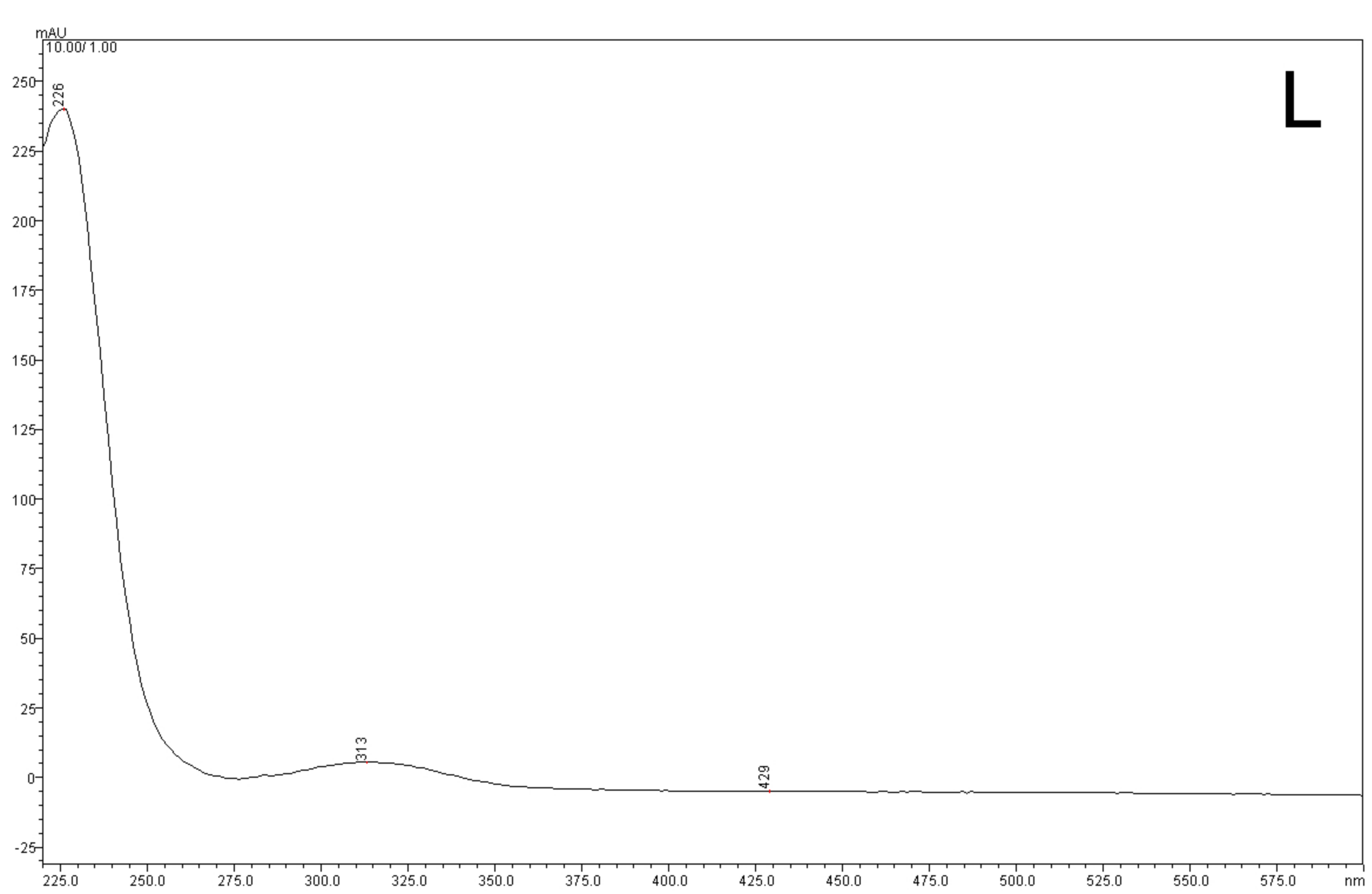

Supplement: Figure S1 — UV absorbance spectra for antimycin A1–A4 and eight antimycin compounds produced by Streptomyces S4. The UV absorbance spectra is shown for A) antimycin A4 (RT = 8.40), B) antimycin A3 (RT = 8.97), C) antimycin A2 (RT = 9.50), D) antimycin A1 (RT = 10.00), E) S4 metabolite 1 (RT = 6.38), F) S4 metabolite 2 (RT = 6.87), G) S4 metabolite 3 (RT = 7.43), H) S4 metabolite 4 (RT = 8.00), I) S4 antimycin A4 (RT = 8.40), J) S4 antimycin A3 (RT = 8.97), K) S4 antimycin A2 (RT = 9.50), L) S4 antimycin A1 (RT = 10.00). (PDF) [file pone.0022028.s001.pdf]

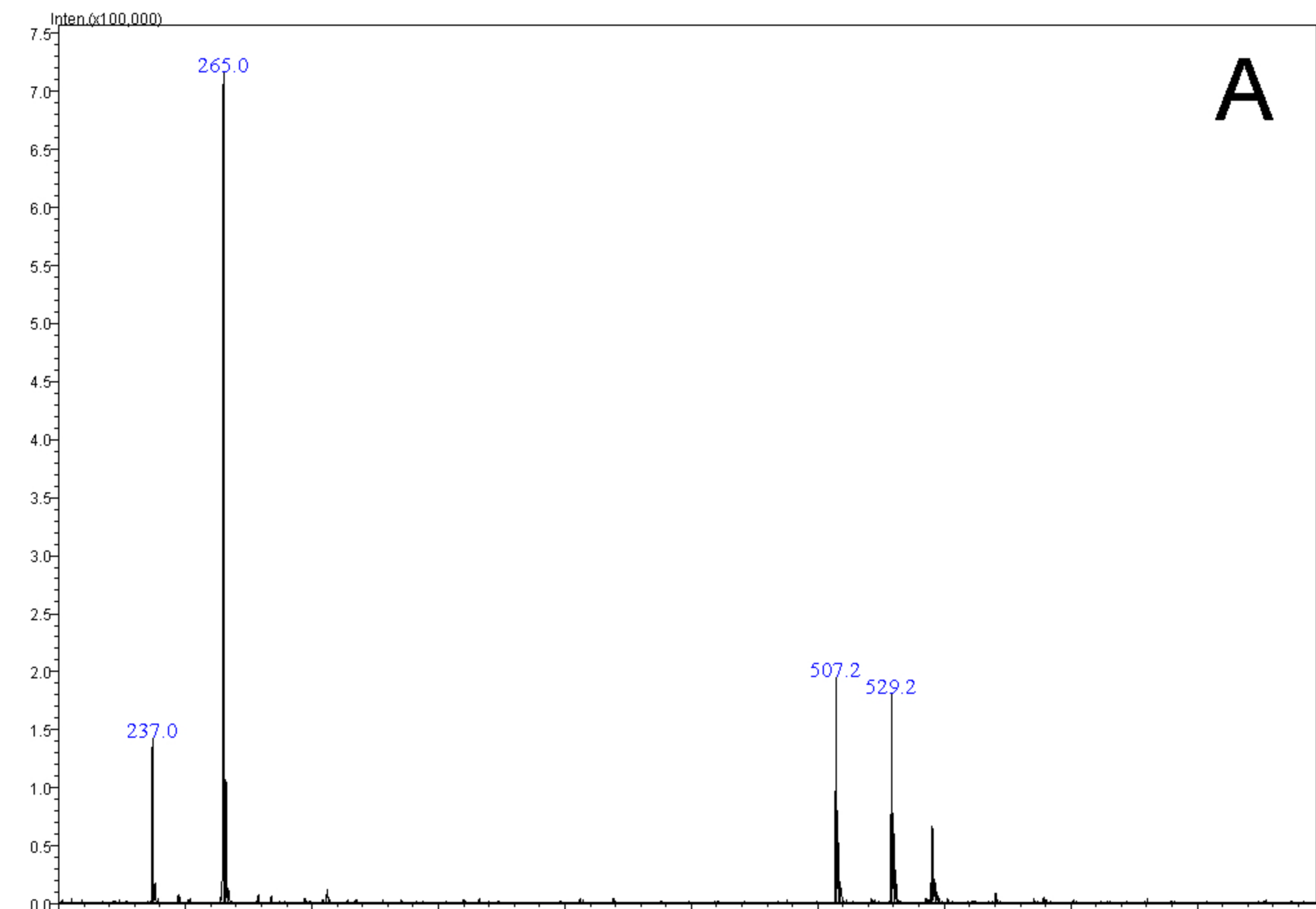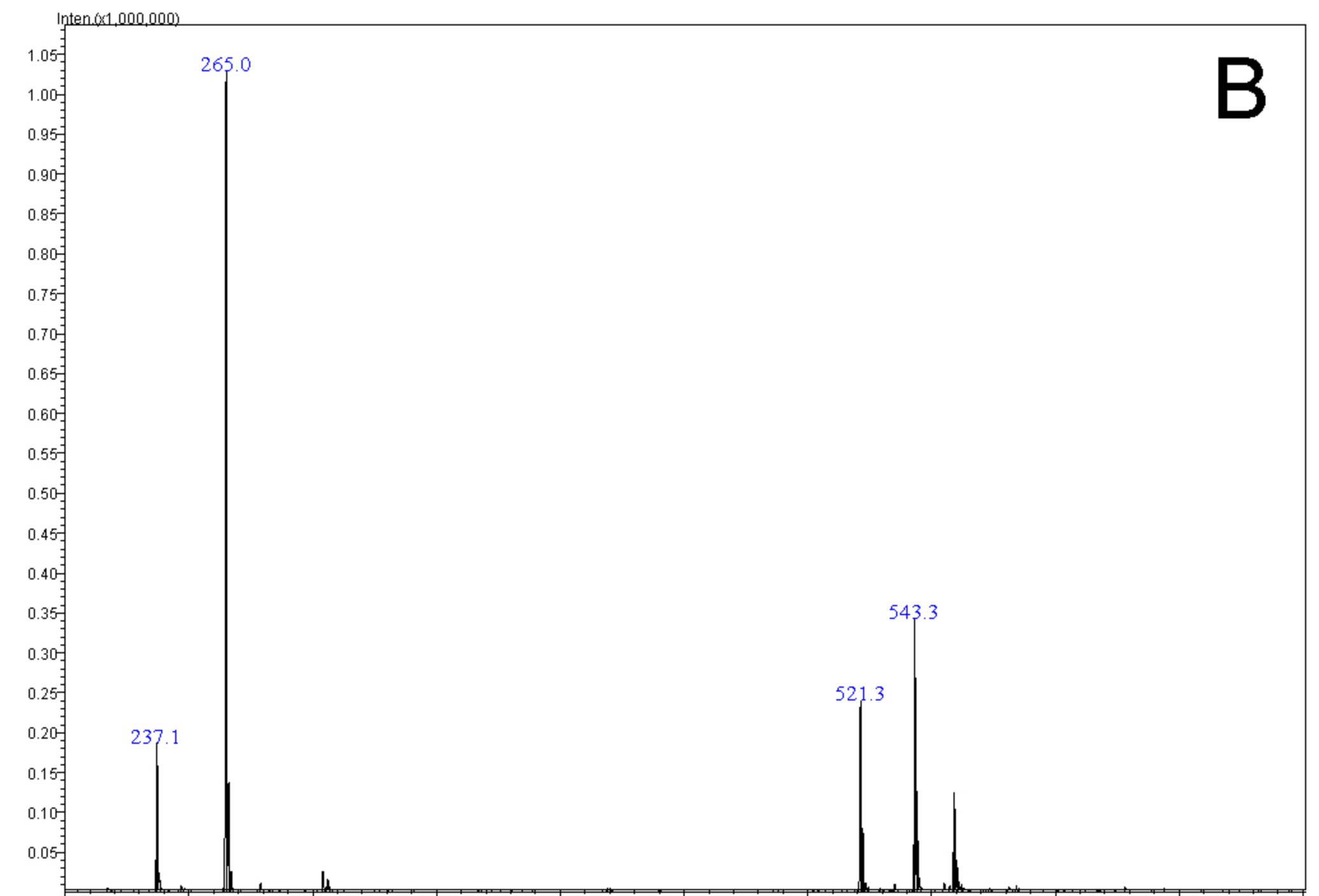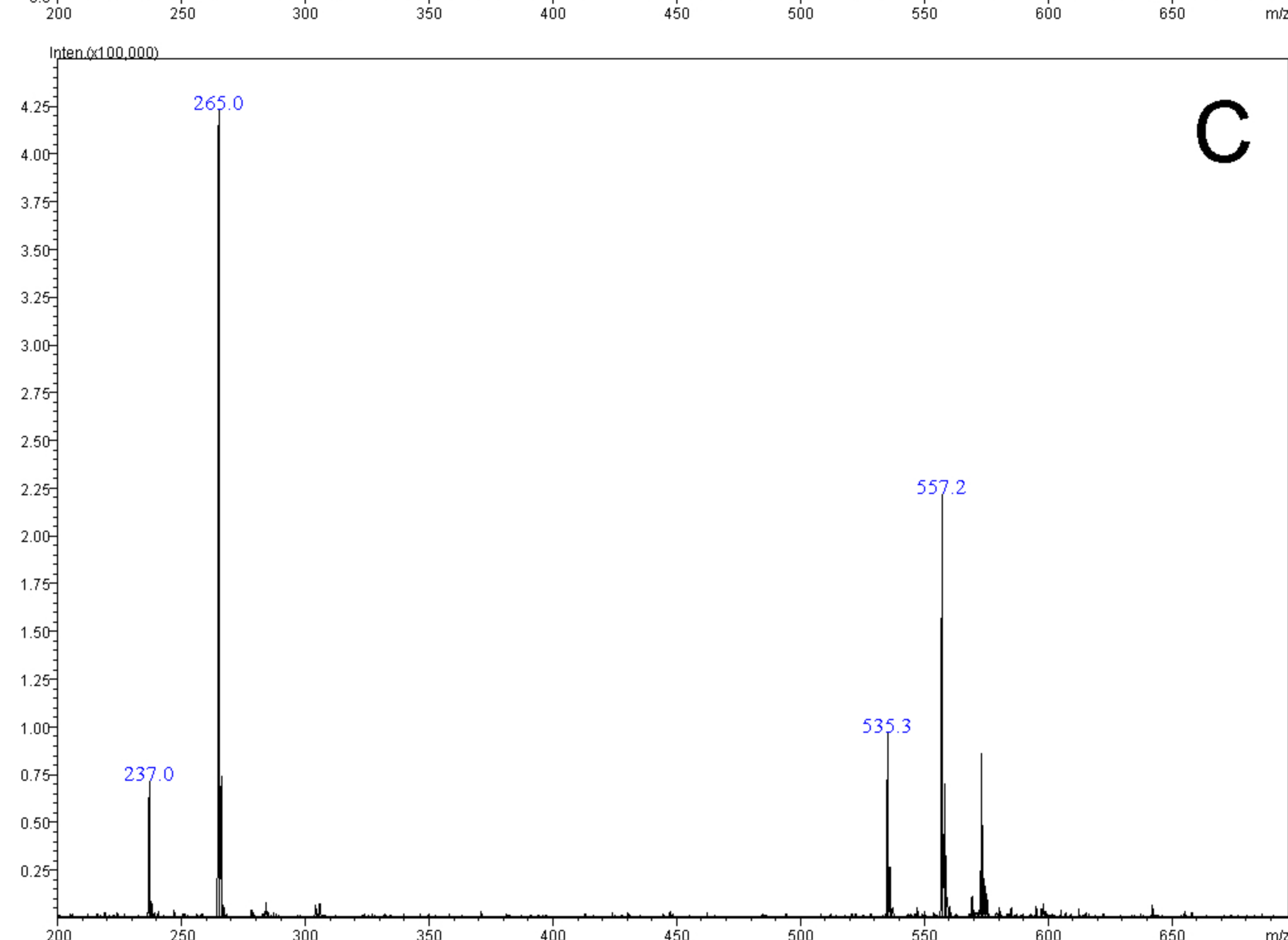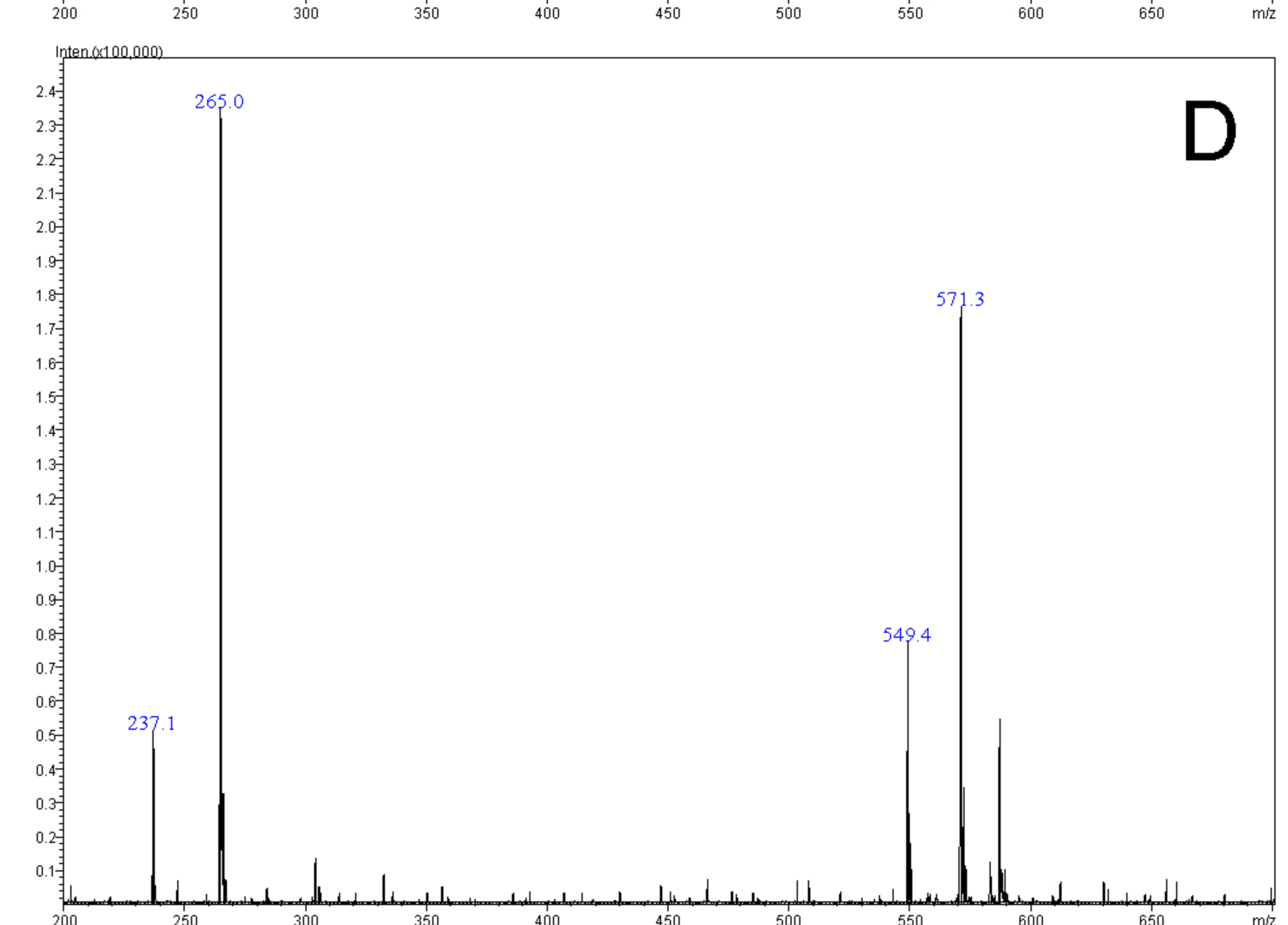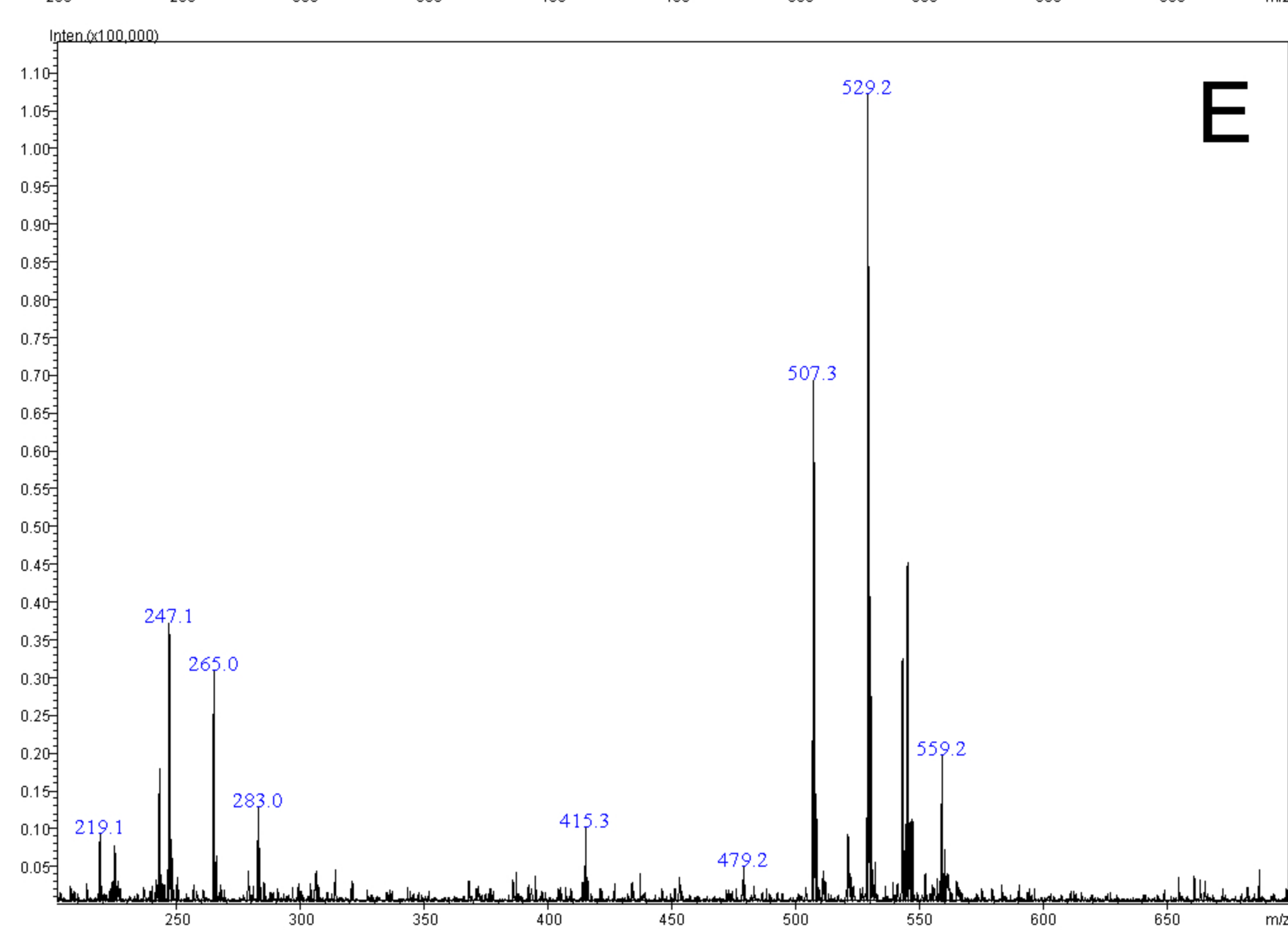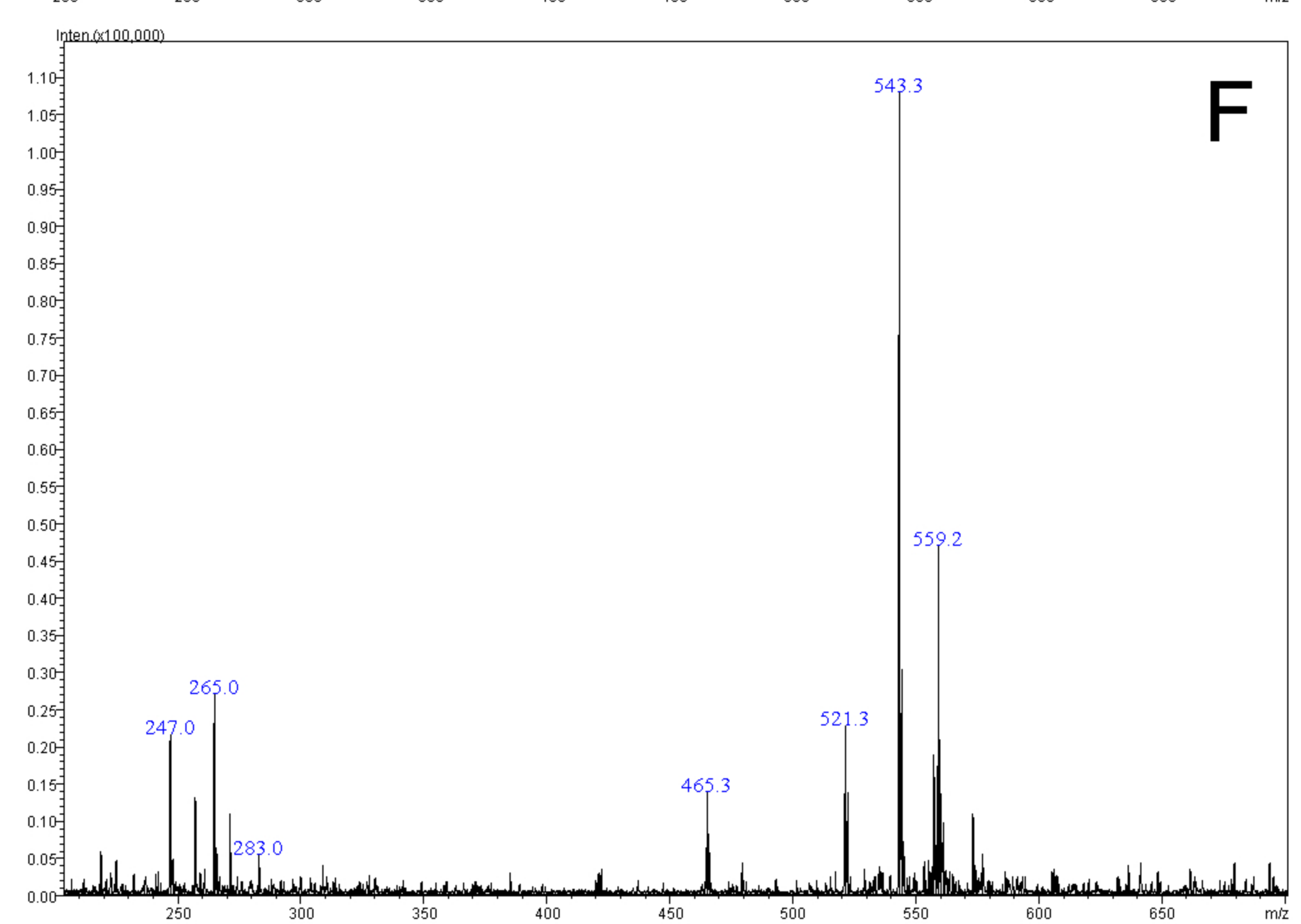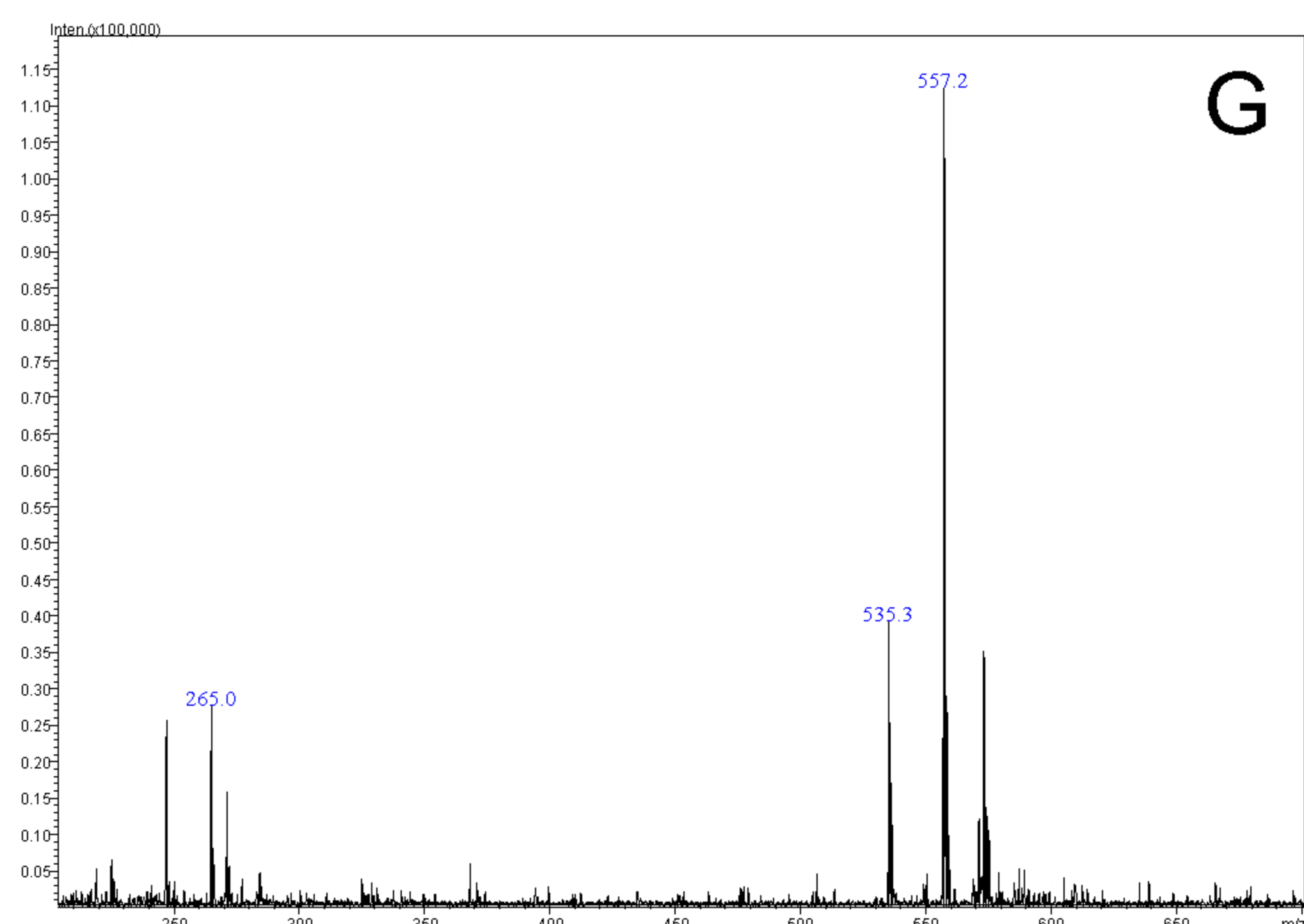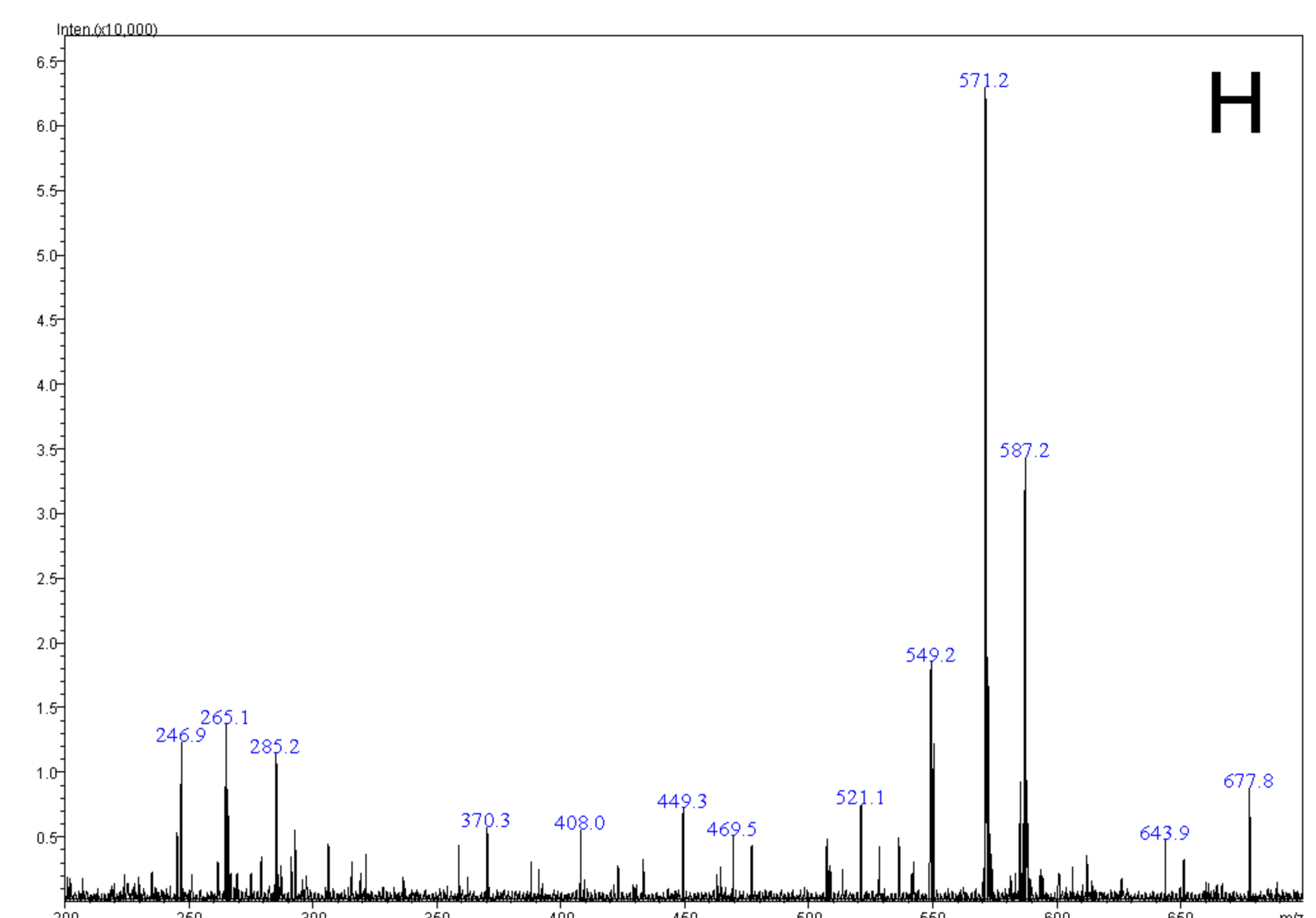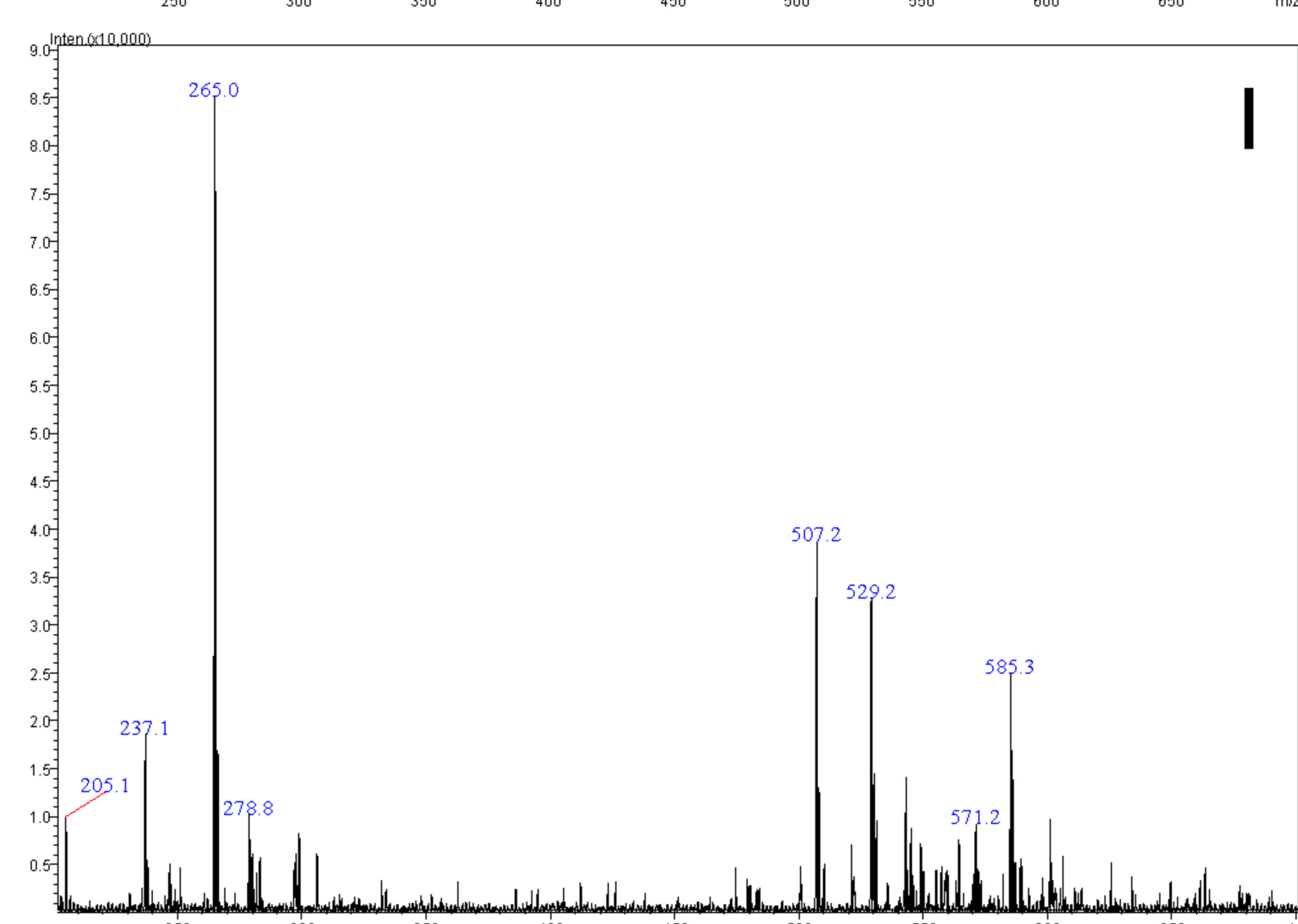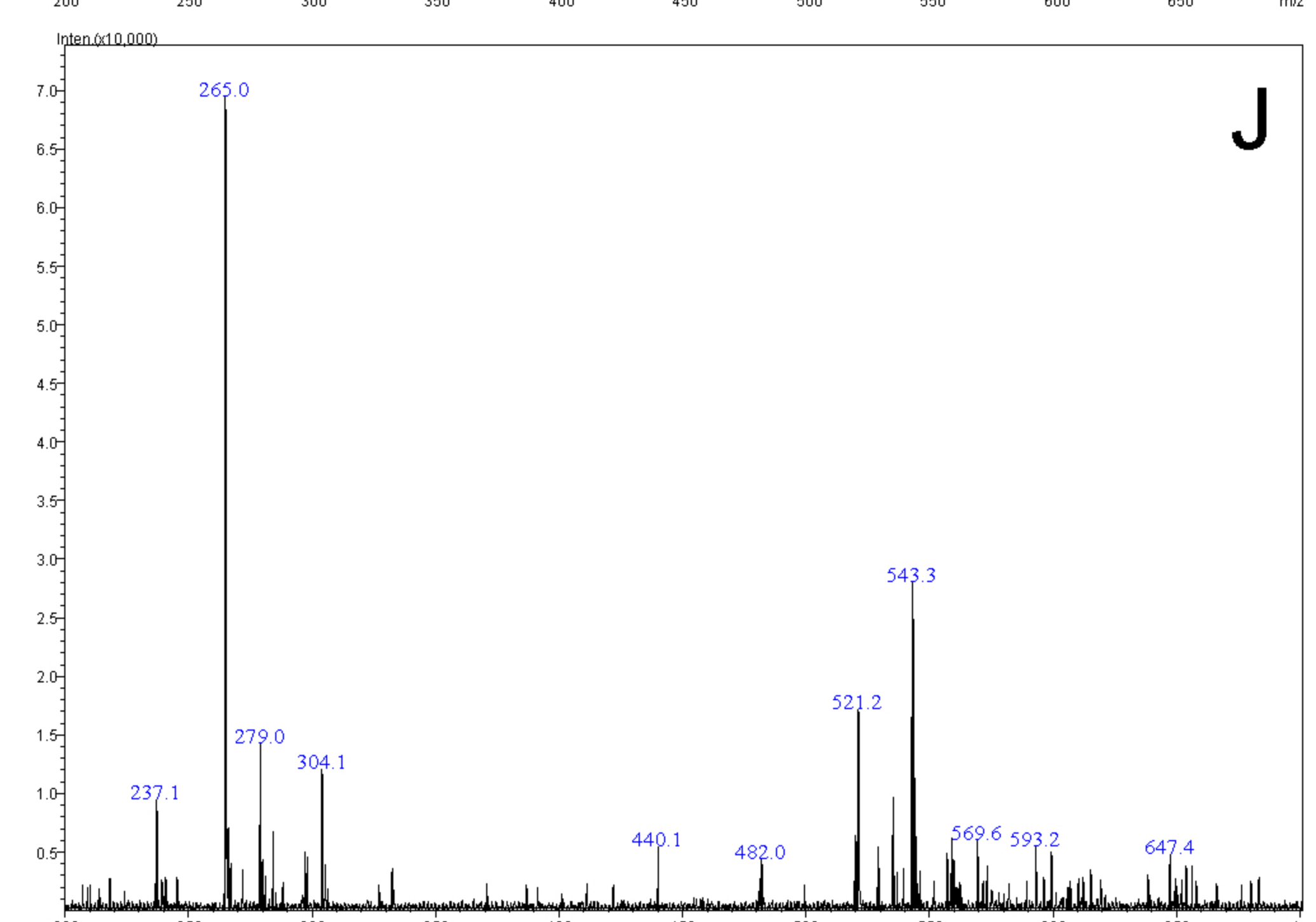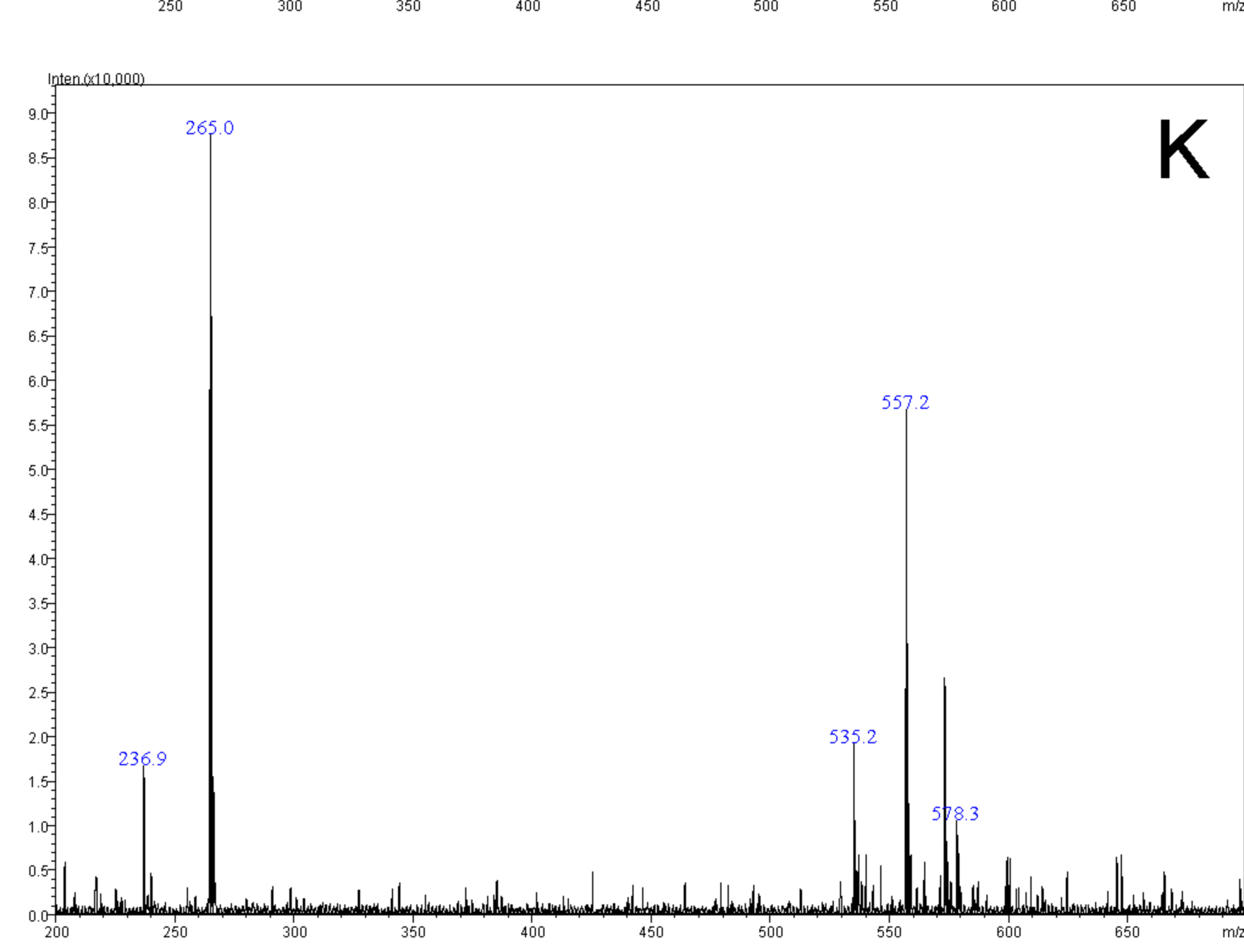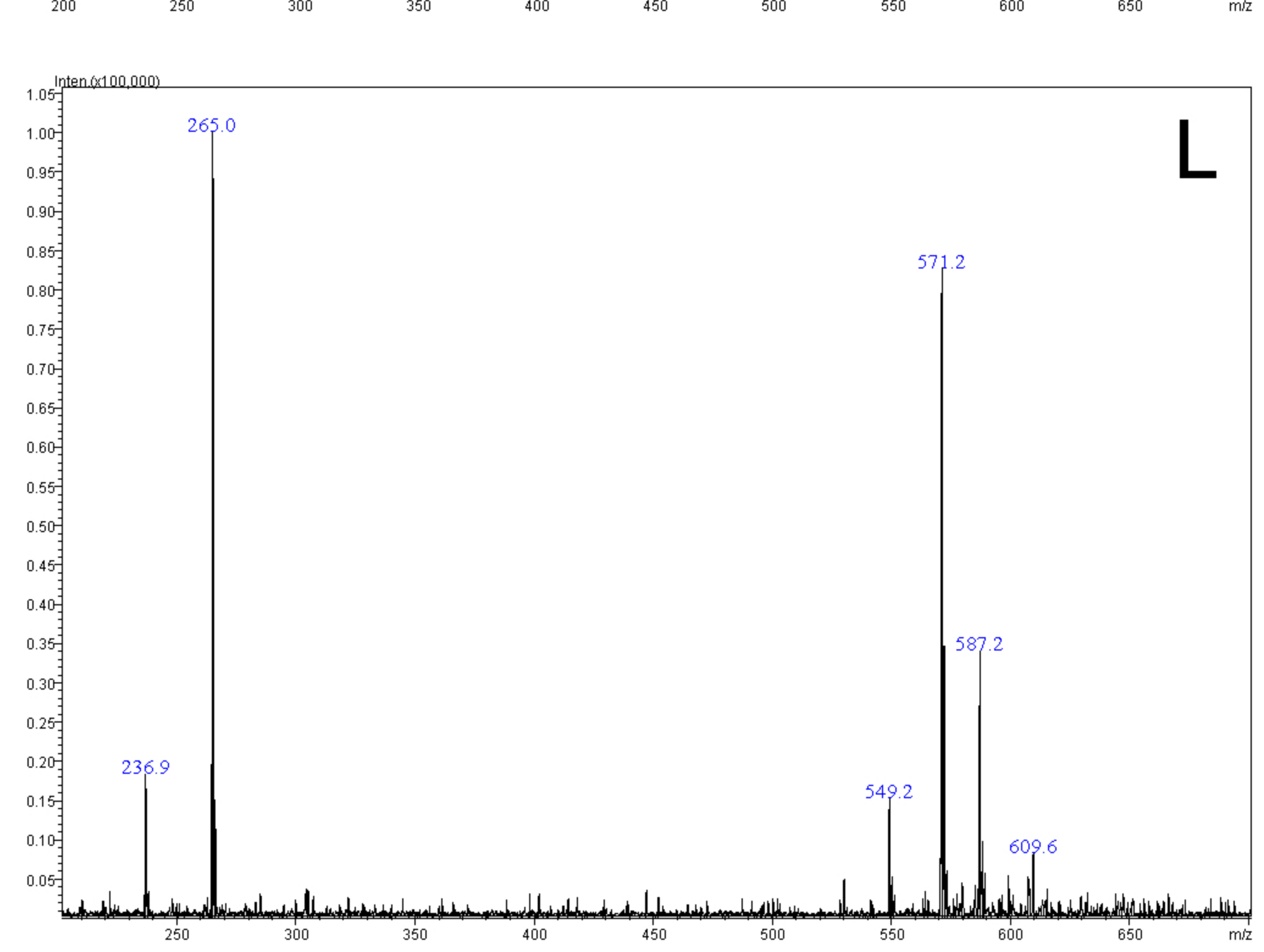

Supplement: Figure S2 — Mass spectra for antimycin A1–A4 and eight antimycin compounds produced by Streptomyces S4. The ESI positive mode detection mass spectra is shown for A) antimycin A4 (RT = 8.40), B) antimycin A3 (RT = 8.97), C) antimycin A2 (RT = 9.50), D) antimycin A1 (RT = 10.00), E) S4 metabolite 1 (RT = 6.38), F) S4 metabolite 2 (RT = 6.87), G) S4 metabolite 3 (RT = 7.43), H) S4 metabolite 4 (RT = 8.00), I) S4 antimycin A4 (RT = 8.40), J) S4 antimycin A3 (RT = 8.97), K) S4 antimycin A2 (RT = 9.50), L) S4 antimycin A1 (RT = 10.00). (PDF) [file pone.0022028.s002.pdf]
