# Supplementary material for: A Single Streptomyces Symbiont Makes Multiple Antifungals to Support the Fungus Farming Ant Acromyrmex octospinosus
Source: PLoS One. 2011 Aug 3;6(8):e22028. doi: 10.1371/journal.pone.0022028 (PMC3153929; doi:10.1371/journal.pone.0022028)
Supplement: Table S1 — Oligonucleotide primers used in this study. (DOC) [file pone.0022028.s003.doc]

**Table S1. Oligonucleotide primers used in this study**

| Name | Sequence (5'-3')* | Description |
| --- | --- | --- |
| RFS78 | ATA**GGATCC**CAGACGTGCCGTGGGTG | PCR: upstream knockout arm for *fscC* deletion, HindIII |
| RFS79 | ATA**AAGCTT**CCGTCAGCACCAGACCAC | PCR: upstream knockout arm for *fscC* deletion, BamHI |
| RFS80 | ATA**GAATTC**TCGGTTCTGGATGGGAC | PCR: downstream knockout arm for *fscC* deletion, EcoRI |
| RFS81 | ATA**GGATCC**ATCATCGGGTGCAGGTC | PCR: downstream knockout arm for *fscC* deletion, BamHI |
| RFS94 | atatctaga**ggatcc**ctgacgccgttggatacaccaagga | PCR: hygromycin B resistance cassette, BamHI |
| RFS95 | atatctaga**ggatcc**tctaaagtatatatgagtaaacttg | PCR: hygromycin B resistance cassette, BamHI |
| RFS115 | cattcttcgcatcccgcct | PCR: internal fragment of the *aac(3)*IV apramycin resistance gene |
| RFS116 | cattcttcgcatcccgcct | PCR: internal fragment of the *aac(3)*IV apramycin resistance gene |
| RFS117 | AGACGGGACGGAGGCATC | PCR: internal fragment of *fscC,* confirmation of *fscC* knockout |
| RFS118 | CGACGGCATGGTCCTCTTC | PCR: internal fragment of *fscC,* confirmation of *fscC* knockout |
| RFS121 | AAA**GGATCC**ACGATCTGGTCCTCGGGCTC | PCR: internal fragment of *antC* gene for disruption mutant, BamHI |
| RFS122 | AAA**GAATTC**CAGCCGGTGGAAGGTGAC | PCR: internal fragment of *antC* gene for disruption mutant, EcoRI |
| RFS147 | acgccgacacggtgagac | PCR: internal fragment of *antC,* confirmation of *antC* disruption |
| RFS148 | cggagcggtacatccggttg | PCR: internal fragment of *antC,* confirmation of *antC* disruption |
| M13F | GTAAAACGACGGCCAG | PCR: internal fragment of *lacZ*α in pGEMT-EZ, confirmation of *antC* disruption |
| M13R | CAGGAAACAGCTATGAC | PCR: internal fragment of *lacZ*α in pGEMT-EZ, confirmation of *antC* disruption |

* engineered restriction endonuclease sites are bolded
